# UHPLC-Q-TOF-MS/MS profiling of resin glycosides and their lipase inhibitory activity in leaves of selected sweet potato (*Ipomoea batatas* L.) cultivars

**DOI:** 10.3389/fnut.2026.1783378

**Published:** 2026-03-19

**Authors:** Yi Lin, Zhixuan Song, Qingtong Xie, Yuyun Lu, Molan Zhang, Joanne Yi Hui Toy, Dejian Huang

**Affiliations:** 1 Department of Food Science and Technology, National University of Singapore, Singapore, Singapore; 2 National University of Singapore (Suzhou) Research Institute, Suzhou, Jiangsu, China

**Keywords:** agriceuticals, cultivars, lipase inhibitory activity, resin glycosides, sweet potato leaves

## Abstract

**Introduction:**

Resin glycosides (RGs) found in the aerial parts of sweet potato are health promoting agents that can be reclaimed from the by-products of sweet potato plantation as agriceuticals for combating obesity. However, RGs have complex structural variations across various sweet potato cultivars, along with an unclear structure–activity relationship. To address this gap, nine types of sweet potato leaves were evaluated, including “Shangshu 19”, “Xuzi 20-1”, “Xushu 24”, “Xushu 32”, “Yanshu 25”, “Blackheart”, “Blackie”, and two “Beniazuma” samples.

**Methods:**

RG-rich extracts were obtained from sweet potato leaves by using dichloromethane extraction. Pancreatic lipase (PL) inhibitory activity was then determined by a p-nitrophenyl palmitate (pNPP)-based assay. Individual RG profiles were characterized and semi-quantified by UHPLC–Q-TOF-MS/MS, followed by multivariate analysis to evaluate cultivar-dependent variations and identify spectral features associated with PL inhibition.

**Results:**

Xushu 32 and Blackheart cultivars exhibited the highest PL inhibitory activities with an Orlistat equivalent (OE) of 2.44 ± 0.30 and 2.02 ± 0.10 ng orlistat/μg extracts, respectively. In total, 128 RGs were tentatively identified based on UHPLC–Q-TOF-MS/MS analysis. Multivariate analysis revealed cultivar-dependent differences in RG profiles and identified four RGs as potential marker compounds associated with PL inhibition. All four RGs were pentasaccharides featuring 2-methylbutyric and dodecanoic acid as side chains. Further mechanistic study revealed that long-chain fatty acid esters or a macrocyclic lactone moiety on the RG backbone may play a key role in conferring inhibition.

**Discussion:**

Taken together, these findings may serve as valuable guidelines for foodomic analysis of RGs and cultivar selection in developing anti-obesity functional foods derived from agricultural by-products.In addition, we would like to double-check that the formatting of Table 1 is maintained as in the original manuscript we provided. The current proof version includes additional internal table lines that slightly alter the intended meaning of the table.

## 1 Introduction

Sweet potato, also known as “camote” in Mexico, is a popular vegetable throughout the world, especially in the Oriental countries. This plant belongs to the Convolvulaceae family and is notable for the presence of a distinctive constituent called resin glycosides (RGs). RGs are a type of complex glycolipids that have gained interest in the field of natural medicine research due to their diverse biological activities (1). To date, hundreds of RGs have been discovered and various activities have been reported, including antimicrobial, antiviral, antimalarial, anti-metastatic, anti-inflammatory, anti-proliferative, neuroprotective, vasorelaxant, and anticancer activities, α-glucosidase inhibition as well as multidrug-resistance modulating properties, indicating their potential as active ingredients in nutraceuticals (1, 2).

Recent research from our group shows the RGs are potent pancreatic lipase (PL) inhibitors, which could potentially be explored for body weight management (3). For instance, RGs derived from *Ipomoea aquatica* demonstrated a dose-dependent inhibitory effect on PL, with an OE value of 6.86 ± 0.51 × 10^−4^ g orlistat/ g extracts (4). Moreover, *in vitro* lipolysis studies revealed that co-consumption of RGs with high-fat foods, such as butter and salad dressing, effectively delayed enzymatic fat digestion by inhibiting PL activity. Notably, incorporating 10.8% RGs resulted in a significant 55.2% ± 4.1% reduction in fat hydrolysis (4). Additionally, our research into sweet potato leaf (SPL) extracts from three different cultivars reveals that their lipase inhibitory activity is notably cultivar-dependent, with OE values ranging from 1.22 ± 0.06 × 10^−4^ to 3.83 ± 0.36 × 10^−4^ g orlistat/ g extracts (3). These findings suggest that the bioactivity of RGs, similar to other phytochemicals, is influenced not only by cultivar type but also by agronomic conditions, underscoring the importance of investigating the structure–activity relationships (SARs) underlying RG-mediated lipase inhibition.

Given the global diversity of sweet potato cultivars, we propose to expand this research to include a broader selection, particularly those popular in China and Singapore, to deepen our understanding in this field. However, the traditional approach to identifying RGs via nuclear magnetic resonance (NMR) spectroscopy remains a significant challenge, primarily due to the highly complex and difficult-to-interpret signals (5). Thus, more effective and comprehensive approaches for annotating and quantifying RGs are of vital importance. Ultra-high-performance liquid chromatography (UHPLC) coupled with quadrupole, hybrid orthogonal acceleration time-of-fight tandem mass spectrometry (Q-TOF-MS/MS), a nascent hyphenated technique, has gained popularity in the analysis of plant metabolites in recent years. The use of UHPLC provides enhanced chromatographic resolution, improved sensitivity, and shorter analysis times due to smaller particle sizes in the chromatographic columns (6). The Q-TOF-MS/MS component of the technique provides accurate mass measurements and fragmentation information, allowing for the identification and characterization of metabolites in complex mixtures. Together, UHPLC/Q-TOF-MS/MS is the method of choice for the rapid and systematic elucidation of the chemical profiles of different plants.

In this study, the UHPLC/Q-TOF-MS/MS was employed to analyze the types and relative contents of RGs in nine different SPL cultivars. Subsequently, principal component analysis (PCA), partial least squares discriminant analysis (PLS-DA) and orthogonal partial least squares-discriminant analysis (OPLS-DA) were used to discrimination, comparing the relative abundance of different types of RGs based on ion intensity. The present research will provide useful information on the anti-obesity and health-promoting characteristics of SPL, optimizing their use in dietary supplements and agriceuticals.

## 2 Materials and methods

### 2.1 Materials

#### 2.1.1 Collection of sweet potato leaf samples

The SPL of Shangshu 19 (Shang19), Xuzi 20–1 (XuZi20-1), Xushu 24 (Xu24), Xushu 32 (Xu32), Yanshu 25 (Yan5) were provided by Xuzhou Sweet Potato Research Institute (Xuzhou, China). The leaves of Blackheart and Blackie, were obtained from Farmily Pte Ltd., a sweet potato farm in Singapore. In addition, two types of SPL from the cultivar *Beniazuma* were obtained from two cultivation systems: The Beniazuma D (BD) sample was cultivated indoors under a controlled hydroponic system, whereas the Beniazuma J (BJ) sample was grown under conventional soil-based conditions. These distinct cultivation environments were selected to evaluate potential environmental effects on RG compositions. To ensure a fair comparison, both groups originated from the same propagation stocks prepared from single-leaf-node cuttings of bedded storage roots obtained from a local supplier (NTUC, Singapore). In the hydroponic system, plants (BD) were grown in a rack-based flood-and-drain system using perlite as the substrate. Fertigation was provided three times daily at 6 a.m., 12 p.m., and 6 p.m. using nutrient solution prepared from 1.6 parts Nutriflex T (NPK 15-8-25): 1part CaCl_2_ by weight, adjusted to an electrical conductivity of 2.0 dS m^−1^ and pH of 5.8. In the soil-based treatment, plants (BJ) were transplanted into pots containing soil. Each treatment included six replicates. Sweet potato leaves were harvested at week 6, consistent with common industrial practice. From each group, five leaves were collected per plant. The collected leaves had an average leaf area of 53.66 ± 3.51 cm^2^ for BD and 52.44 ± 4.12 cm^2^ for BJ. Samples were collected on 10 December 2021, pooled, and subsequently freeze-dried. The lyophilized leaves were homogenized using a blender and stored at −20 °C for subsequent analysis.

#### 2.1.2 Chemical reagents

Tris-borate-ethylenediaminetetraacetic acid (Tris-Borate-EDTA (TBE)) buffer (10X, 890 mM) was obtained from Vivantis Technologies (Subang Jaya, Selangor, Malaysia). Additionally, other reagents such as lipase (Type II, 100–500 units/mg, L3126 from porcine pancreas), Orlistat (PHR1445), *p*-nitrophenyl palmitate (*p*NPP) (N2752), and sodium deoxycholate (D6750) were purchased from Sigma-Aldrich (St Louis, Mo, USA). Analytical grade solvents, including dichloromethane (DCM), hexane, ethyl acetate (EA), isopropanol and methanol, were supplied by Avantor, Inc. (Radnor Township, Pennsylvania, United States).

### 2.2 Extraction and enrichment of RG

The RGs were extracted from SPLs by using a reported protocol from our group with minor modification (3). Briefly, lyophilized SPL (100 g) were ground and macerated in DCM for 12 h. The liquid fraction was subsequently concentrated to obtain DCM extracts, followed with purification in methanol by using solid–liquid extraction. Then the supernatant was dried at 55 °C with a rotary evaporator, which was further purified using silica gel column sequentially eluted with hexane-EA (5:1) (v/v), hexane-EA (1:1) (v/v), EA and methanol. The eluates in methanol fraction were collected and concentrated for subsequent analysis.

### 2.3 Pancreatic lipase (PL) inhibition assay

The PL inhibition assay was performed by following a reported *p*NPP assay with a minor modification (3). The assay solution consisted of 150 μL SPL sample solution, 10 μL lipase solution and 10 μL *p*NPP solution. The sample solutions were prepared by dissolving the SPL extract in methanol at a concentration of 20 mg/mL, and further diluted by TBE buffer (50 mM, containing 0.35% w/v sodium deoxycholate, pH 8.3) to appropriate concentrations (50–200 μg/mL). The PL solution was prepared by dissolving PL (1.0 mg/mL) TBE buffer solution before centrifugation at 13751 × *g* for 5 min at 4 °C. *p*NPP solution (1.5 mg/mL) was prepared by using isopropanol. In a 96-well microplate (Corning, clear polystyrene, USA), 10 μL of the PL solution was mixed with 150 μL of the SPL sample solution with series concentrations (or 150 μL of TBE buffer solution as negative control, 150 μL of Orlistat solution as positive control). After 15 min incubation at 37 °C, the reaction was initiated by injection 10 μL of *p*NPP solution. The absorbance was immediately monitored at 410 nm every minute for 1 h to determine the reaction rate. The slopes of sample and negative control were used to calculate inhibitory percentage (Equation1):


$$\text{Inhibition}\;(\%)=1-({\text{Slope}}_{\text{sample}}/{\text{Slope}}_{\text{control}})\times 100\%$$ (1)

The IC_50_ (μg/mL), which refers to the inhibitor concentration required to inhibit 50% of enzyme activity under the specified assay condition, was determined as well. To avoid run-to-run variation induced by fluctuation of PL, the Orlistat equivalence (OE) was used to express the inhibitory capacity of samples based on the Equation (2):

(2) $$\mathrm{OE}={\mathrm{IC}}_{50}\;(\text{Orlistat})/{\mathrm{IC}}_{50}\;(\text{Sample})$$

In the kinetic study, PL activity was tested at different concentrations of pNPP substrate in the presence of a series of inhibitor concentrations (4.9–7.4 μM). For each reaction, Initial reaction rates (v_0_) were determined from the linear portion of the absorbance–time curves.

The enzyme kinetics were then evaluated by constructing double-reciprocal Lineweaver–Burk plots (1/v_0_ versus 1/[pNPP]) to determine the mode of inhibition. K_m_ (Michaelis–Menten constant) and V_max_ (maximum reaction rate) values were also calculated from the Lineweaver–Burk plot.

### 2.4 UHPLC-QTOF-MS/MS analysis of RG

SPL extracts obtained in “section 2.2” were dissolved in methanol to give a 1 mg/mL solution, which was filtered through a 0.45 μm PTFE syringe filter membrane to obtain filtrate that was ready for injection. Extraction replicates were prepared using the same extracts, which were similarly purified, concentrated, suspended and filtered, to obtain another set of filtrate ready for injection. A quality control (QC) sample was prepared by combining equal aliquots from each sample.

Samples were separated by using the ACQUITY UPLC™ H-Class PLUS system (Waters, Milford, MA, USA), with an ACQUITY UPLC C18 column (2.1 × 100 mm, 1.8 μm; Waters, Singapore) at 30 °C. The flow rate was set at 0.4 mL/min and the injection volume was 1 μL. The mobile phase consisted of 0.1% formic acid (FA) in water (Honeywell Burdick & Jackson, Muskegon, MI, USA) (A) and 0.1% FA in methanol (Honeywell Burdick & Jackson, Muskegon, MI, USA) (B), and gradient conditions were as follows: 0–7 min 96% B, 7–8 min 96–100% B, 8–15 min 100% B, 15–15.1 min 100–96% B, 15.1–20 min 96% B. Mass spectrometry (MS) analysis was conducted on a Xevo G2-XS quadrupole time-of-flight mass spectrometer (QTOF-MS) (Waters, Manchester, U. K.) adopted from a reported method with minor modifications (7). The data were acquired under negative ion mode of an electrospray ionization (ESI) source with a continuum mode. The measurement conditions for mass spectrometry were set as follows: the capillary voltage: 2.5 kV; source temperature:120 °C; desolvation temperature: 500 °C; desolvation gas flow:800 L/h; mass range: 50–2,000 Da; scan time: 0.2 s; ramp collision energy (high energy): 20–40 ev.

### 2.5 Data processing and multivariate statistical analysis

The UHPLC–MS spectra were processed using the Progenesis QI software (V.3.0, Nonlinear Dynamics, Waters, Newcastle, UK), on which baseline filtering, retention time alignment, 3D peak picking and response normalization were automatically performed (7). For characterization, we constructed a small RG library of 244 reported RGs in literature. The information in the library include compound names, molecular formula, exact molecular weights, and structure based on SciFinder database^1^ (Supplementary Table S1). The library was saved as .mol format files and integrated into a .sdf format file using Progenesis SDF Studio (Nonlinear Dynamics, Newcastle, UK), which was further imported into Progenesis QI software as reference library for RG identification. In addition, abundances of RGs were determined automatically in Progenesis QI based on the sum of the ion intensities within the isotope boundaries, providing semi-quantitative measurements for relative comparisons across samples. Furthermore, PCA, PLS-DA and OPLS-DA of the spectral data were performed on the EZinfo software (V.3.0, Umetrics, Sweden). Subsequently, the significantly altered RGs in each pairwise analyses were filtered out based on variables important in projection (VIP) > 1, *p* value < 0.05 and CV ≤ 30.

## 3 Results and discussion

### 3.1 RG profiling and multivariate statistical analysis based on UPLC-QTOF-MS/MS

RGs are secondary metabolites present in numerous plant species, particularly in the Convolvulaceae family (5). In this study, one hundred and twenty-eight (128) RGs were putatively identified from different sweet potato leaves, including 1 trisaccharide, 33 tetrasaccharides, and 94 pentasaccharides. The total ion chromatogram (TIC) and representative mass spectra of RGs are shown in Supplementary Figures S1, S2, respectively. In addition, detailed information on their retention time, exact mass, molecular formula, precursor type and compound name were provided in Supplementary Table S2.

**Figure 1:**
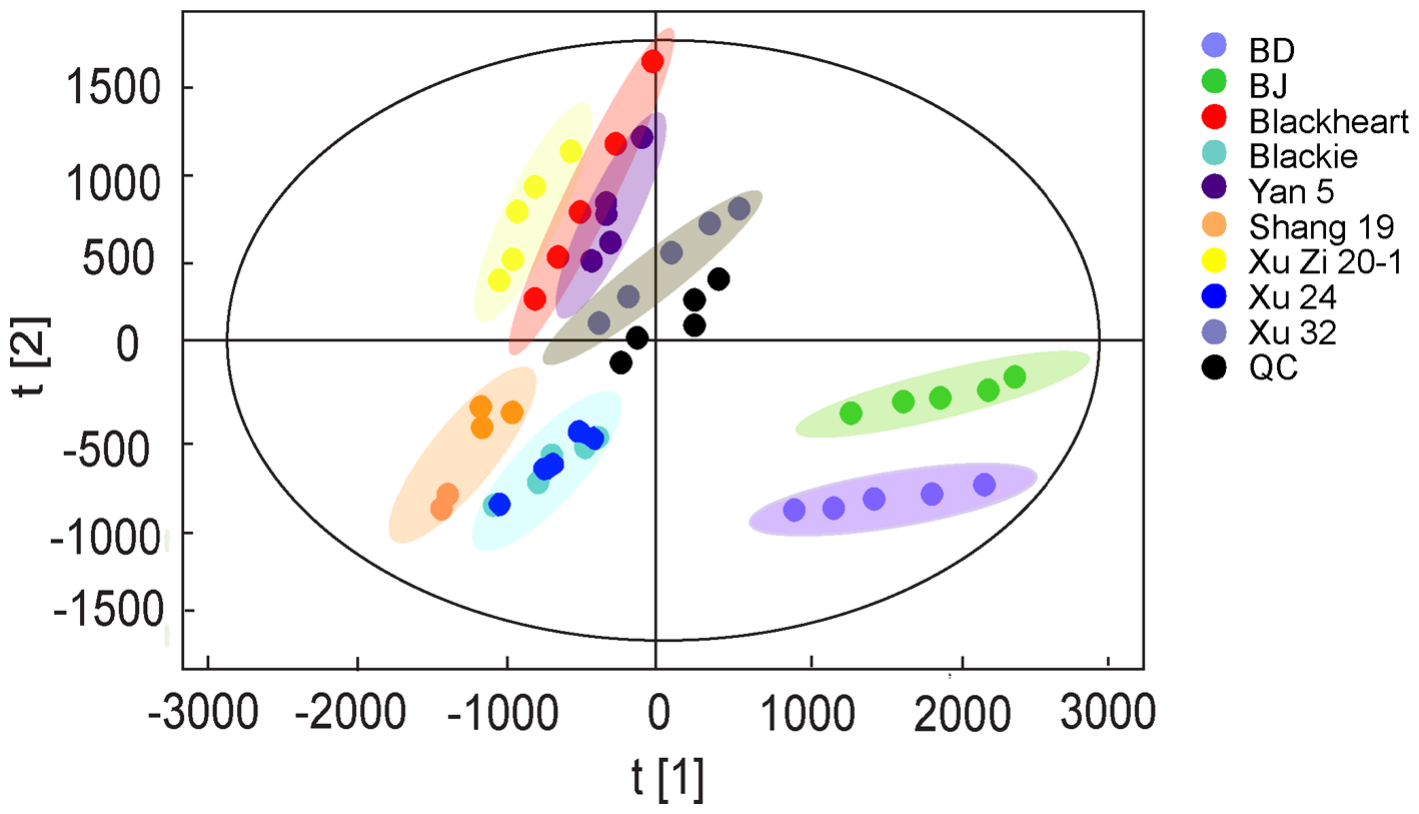
Principal component analysis (PCA) score plots of resin glycosides in sweet potato leaves samples.

To achieve an overview of the RG differences among leaf samples, the unsupervised multivariate data analysis of PCA was conducted as shown in Figure 1. The first and second principal components (PC1 and PC2) showed 47.48% and 16.69% of the variance, respectively. Along the PCA loading plot, QC samples were tightly clustered at the origin of the score plots, indicating good repeatability and robustness of the proposed method. The leave samples were distinctly categorized into three main groups: BJ and BD are located in the lower right quadrant of the score plot, which completely discriminated and separated them from the other samples. In contrast, Blackie, Xu24, Shang19 are grouped in the lower left quadrant, with Xu24 and Blackie showing considerable overlap, while Shang19 is distinctly separated from them, suggesting that they share similar overall RG profiles, albeit with notable differences in specific key compounds. Lastly, Xu32, Blackheart, Xuzi20-1, Yan5 are clustered in the upper left quadrant.

### 3.2 Identification of marker RGs resulting in the classification of different varieties

**Figure 2:**
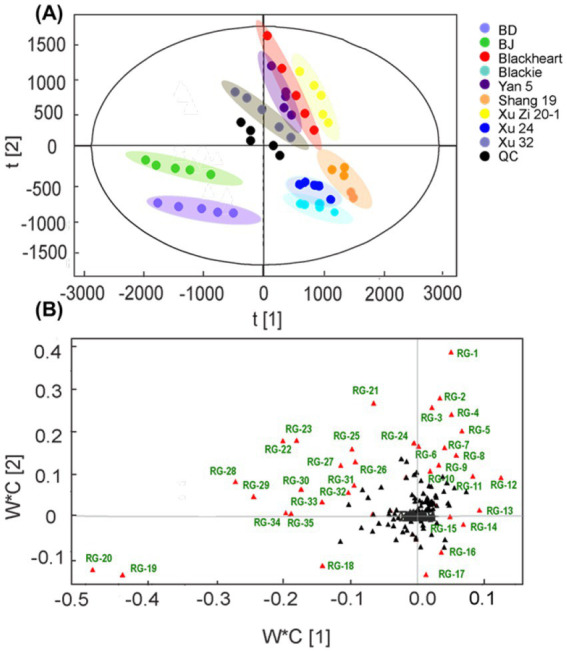
PLS-DA score plot **(A)** and loading plot **(B)** of resin glycosides in sweet potato leave samples. Red triangles represent resin glycosides (RGs) with variable importance in projection (VIP) values > 1 and *p* < 0.05, which were considered the most discriminant metabolites.

To further understand RG characteristics in these SPLs, PLS-DA analysis was performed. As shown in Figure 2A, all samples were separated into three distinct clusters by PLS-DA: Blackie, Xu24 and Shang19 were located in the lower right quadrant, whereas BD and BJ were grouped in the lower left quadrant. The remaining cultivars were distributed in the upper right quadrant. This clustering pattern was consistent with that observed in the PCA analysis. Discriminative RGs were identified based on VIP > 1.0 and *p* < 0.05 in the PLS-DA model, as shown in the loading plot (Figure 2B). In total, 35 RGs, including 4 tetrasaccharides and 31 pentasaccharides, were selected (Table 1). In general, these RGs consist of an oligosaccharide core, a long-chain fatty acid aglycone, and modified acyl groups. Specifically, the oligosaccharide core comprises 4–5 sugar units derived from monosaccharides such as _D_-glucose, _L_-rhamnose, _D_-fucose, _D_-quinovose, and _D_-xylose.11S-Hydroxyhexadecanoic acid (jalapinolic acid) serves as the aglycone, folding back to the oligosaccharide core to form a macrolactone ring through either type A or type B linkages. The acyl modifications vary and include short-chain aliphatic acids such as acetic (Ac), propionic (Pa), isobutyric (Iba), 2R-methylbutyric (2-mba), and angelic acid (Aga); long-chain fatty acids, e.g., *n*-octanoic (Octa), and *n*-decanoic (Deca), *n*-dodecanoic acid (Dodeca); and an arylalkyl acid, trans-cinnamic acid (Cna). It is worth noting that some peaks have multiple assignments due to the complexity of RG structures and the limitations of UPLC-Q-TOF-MS/MS in resolving certain isomers. For example, the method cannot differentiate acyl group modifications occurring at different sites within the same sugar residue, nor can it distinguish diastereomers such as _L_-rhamnose and _D_-fucose. Nonetheless, UPLC-Q-TOF-MS/MS remains a powerful tool, offering a rapid and comprehensive overview, and facilitating efficient characterization and analysis (8).

**Table 1:** Key resin glycosides with VIP > 1.0.

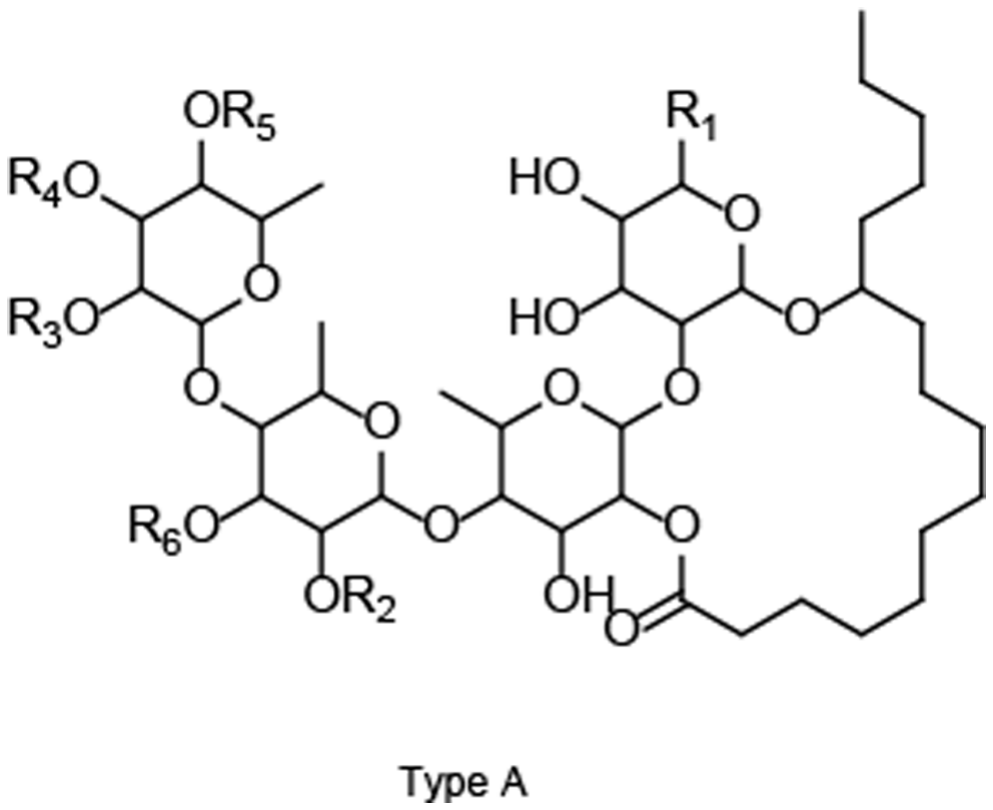 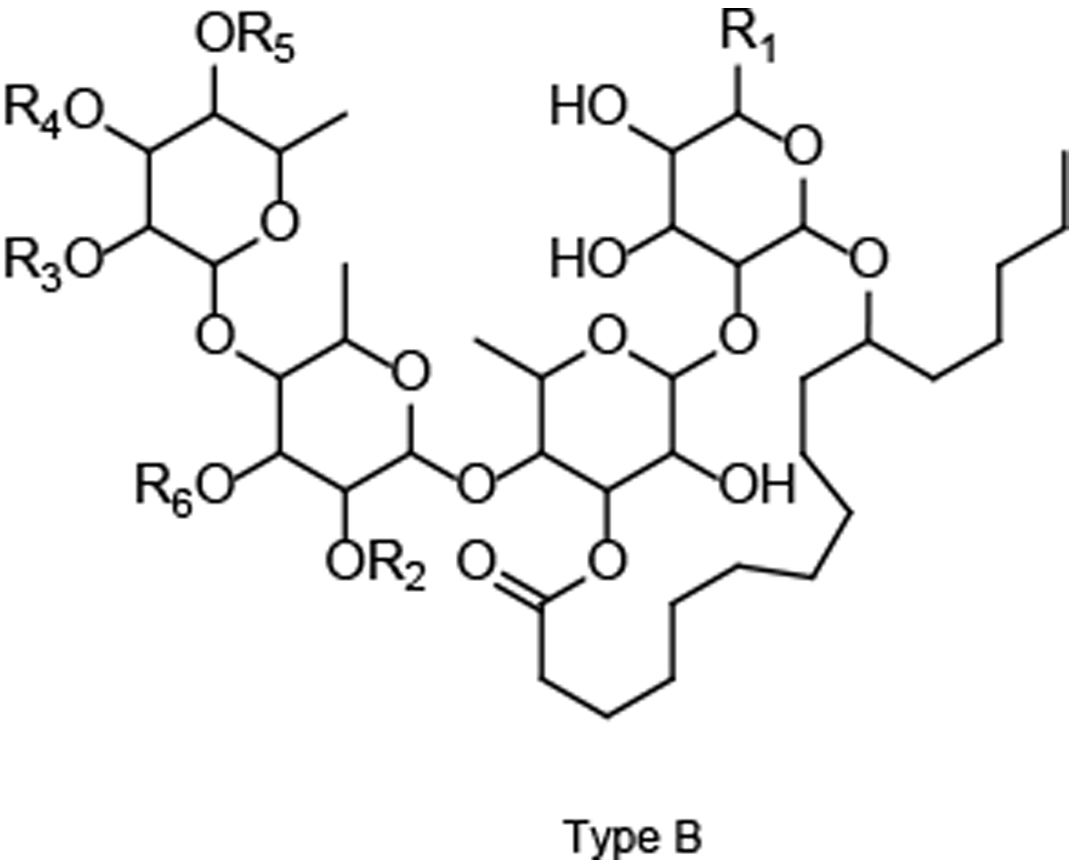												
No	RT (min)	m/z	Formula	Adducts	Compound	Type	R_1_	R_2_	R_3_	R_4_	R_5_	R_6_
RG-1	9.82	1441.7764	C_72_H_116_O_26_	M−H, M+FA−H	Batatoside I	A	CH_3_	2-mba	Cna	H	Dodeca	Glu
RG-29	10.38	1441.7799	C_72_H_116_O_26_	M−H, M+FA−H	Batatoside H	A	CH_3_	2-mba	H	Cna	Dodeca	Glu
RG-3	9.74	1295.7391	C_63_H_110_O_24_	M−H, M+FA−H	Batatinoside VIII	B	CH_3_	Dodeca	H	2-mba	H	Rha
					Pescaprein III	B	CH_3_	Dodeca	H	H	2-mba	Rha
					Stoloniferin X	A	CH_3_	Dodeca	H	H	2-mba	Rha
RG- 7	9.00	1383.7384	C_69_H_110_O_25_	M+FA−H	Batatoside G	A	CH_3_	Dodeca	H	Cna	Ac	Rha
RG-9	8.89	1413.7463	C_70_H_112_O_26_	M+FA−H	Cairicoside A	B	CH_2_OH	Deca	H	Cna	2-mba	Rha
RG-26	9.56	1413.7477	C_70_H_112_O_26_	M−H, M+FA−H	Cairicoside IV	A	CH_2_OH	Deca	H	Cna	2-mba	Rha
					Cairicoside B	B	CH_2_OH	Deca	Cna	H	2-mba	Rha
RG-10	4.03	1313.6641	C_64_H_100_O_25_	M+FA−H	Batatoside A	B	CH_3_	2-mba	Cna	Iba	H	Rha
					Batataoside I	B	CH_3_	2-mba	H	Cna	Iba	Rha
					Batataoside II	A	CH_3_	2-mba	H	Cna	Iba	Rha
RG-13	4.05	1483.8284	C_75_H_122_O_26_	M−H, M+FA−H	Intrapilosin VII	A	CH_3_	Dodeca	H	Cna	Octa	Glu
RG-21	10.18	1425.7796	C_72_H_116_O_25_	M−H, M+FA−H	Batatoside C/ Batatoside E	A	CH_3_	2-mba	Cna	Dodeca	H	Rha
RG-24	10.31	1425.7854	C_72_H_116_O_25_	M−H, M+FA−H	Batatoside B/ Batatoside F	A	CH_3_	2-mba	Cna	Dodeca	H	Rha
					Batatoside D	A	CH_3_	2-mba	H	Cna	Dodeca	Rha
					Batatinoside I	B	CH_3_	2-mba	H	Cna	Dodeca	Rha
RG-22	8.53	1385.7152	C_68_H_108_O_26_	M+FA−H	Cairicoside D	B	CH_2_OH	Octa	H	Cna	2-ma	Rha
					Cairicoside III	A	CH_2_OH	Octa	H	Cna	2-mba	Rha
RG-27	9.30	1383.7409	C_69_H_110_O_25_	M+FA−H	Batatoside P	A	CH_3_	Deca	Cna	H	Iba	Rha
RG-28	10.25	1441.7800	C_72_H_116_O_26_	M−H, M+FA−H	Intrapilosin IV/ Intrapilosin V	A	CH_3_	Dodeca	H	Cna	2-ma	Glu
					Intrapilosin VI	A	CH_3_	Dodeca	H	2-mba	Cna	Glu
RG-31	10.09	1427.7670	C_71_H_114_O_26_	M+FA−H	Intrapilosin III	A	CH_3_	Octa	H	Cna	Octa	Glu
RG-35	9.76	1413.7485	C_70_H_112_O_26_	M+FA−H	Leptophyllin A	A	CH_3_	Dodeca	Cna	H	Pa	Glu

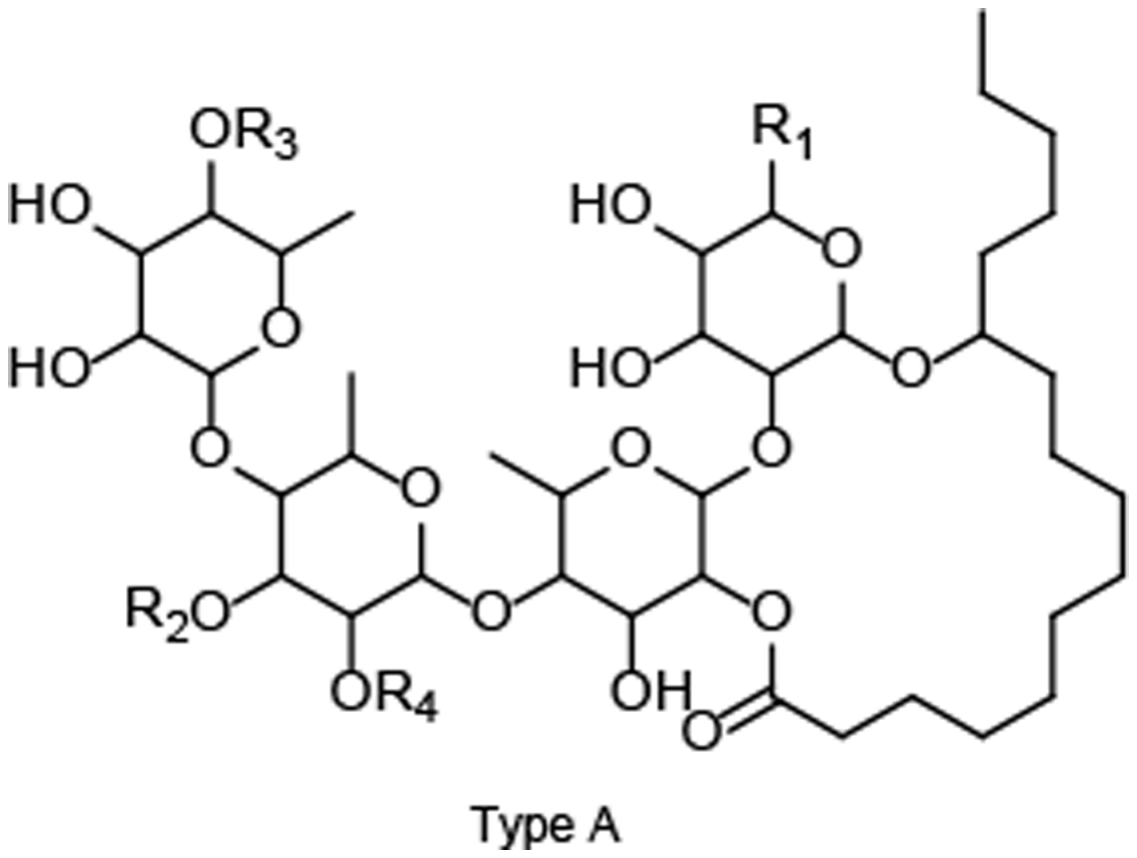 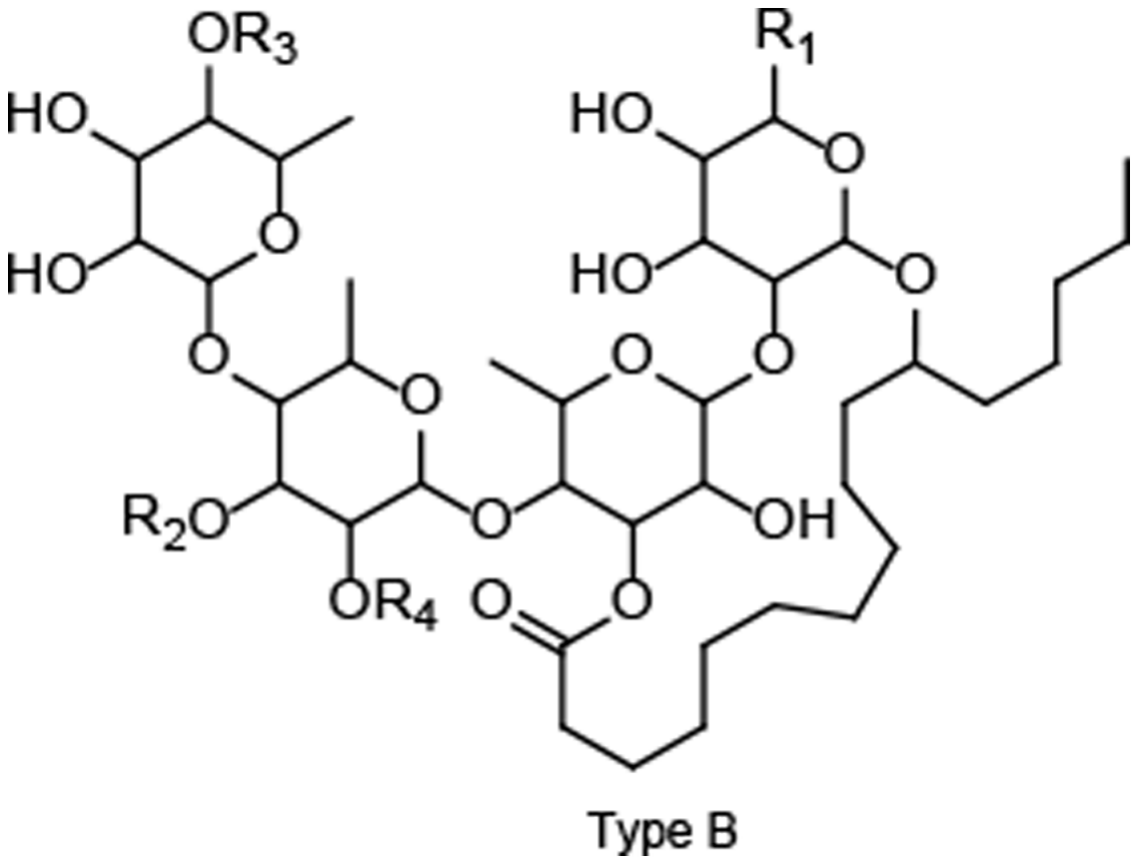										
No	RT (min)	m/z	Formula	Adducts	Compound	Type	R_1_	R_2_	R_3_	R_4_
RG-2	9.28	1311.7347	C_63_H_110_O_25_	M−H, M+FA−H	Cairicoside F	B	CH_2_OH	Dodeca	2-mba	Rha
					Murucoidin VI	B	CH_2_OH	Dodeca	2-mba	Rha
RG-4	10.90	1365.8135	C_68_H_120_O_24_	M−H, M+FA−H	Simonin IV	A	CH_3_	Deca	Dodeca	Rha
RG-5	2.62	1381.8043	C_68_H_120_O_25_	M−H, M+FA−H	Operculin VII	A	CH_3_	Dodeca	Deca	Glu
RG-23	11.11	1381.8156	C_68_H_120_O_25_	M−H, M+FA−H	Stoloniferin I/Operculin VIII	A	CH_3_	Deca	Dodeca	Glu
RG-6	3.88	1255.6727	C_59_H_102_O_25_	M−H, M+FA−H	Quamoclin III	A	CH_3_	Deca	2-mba	Glu
RG-20	5.61	1255.6722	C_59_H_102_O_25_	M−H, M+FA−H	Multifidin I	A	CH_3_	Deca	2-mba	Glu
RG-8	2.99	1397.8066	C_68_H_120_O_26_	M−H, M+FA−H	Operculin IX	A	CH_2_OH	Dodeca	Deca	Glu
					Operculin X	A	CH_2_OH	Deca	Dodeca	Glu
RG-14	2.44	1183.6231	C_55_H_94_O_24_	M+FA−H	Murucoidin IX	B	CH_3_	2-mba	Iba	Rha
					Murucoidin II	A	CH_3_	2-mba	Iba	Rha
RG-16	5.73	1239.6762	C_59_H_102_O_24_	M−H, M+FA−H	Stoloniferin VIII	A	CH_3_	Octa	2-mba	Rha
RG-34	5.12	1239.6773	C_59_H_102_O_24_	M−H, M+FA−H	Murucoidin XVIII	B	CH_3_	Octa	2-mba	Rha
RG-17	3.63	1213.6298	C_56_H_96_O_25_	M+FA−H	Murucoidin IV	A	CH_3_	2-mba	2-mba	Glu
RG-18	2.98	1197.6352	C_56_H_96_O_24_	M−H, M+FA−H	Murucoidin III	A	CH_3_	2-mba	2-mba	Rha
RG-19	8.57	1283.7032	C_61_H_106_O_25_	M−H, M+FA−H	Cairicoside E	B	CH_2_OH	Deca	2-mba	Rha
RG-25	9.87	1311.7349	C_63_H_110_O_25_	M−H, M+FA−H	Simonin II	A	CH_2_OH	2-mba	Dodeca	Rha
RG-30	10.49	1353.7847	C_66_H_116_O_25_	M−H, M+FA−H	Operculin II	A	CH_3_	Deca	Deca	Glu
RG-32	9.25	1283.7047	C_61_H_106_O_25_	M+FA−H	Stoloniferin IV	B	CH_3_	Deca	2-mba	Glu
					Stoloniferin V	A	CH_3_	Deca	2-mba	Glu
RG-33	7.80	1267.7093	C_61_H_106_O_24_	M−H, M+FA−H	Pescaprein XXX/Stoloniferin IXX	A	CH_3_	Deca	2-mba	Rha
					Stoloniferin III	B	CH_3_	Octa	2-mba	Rha

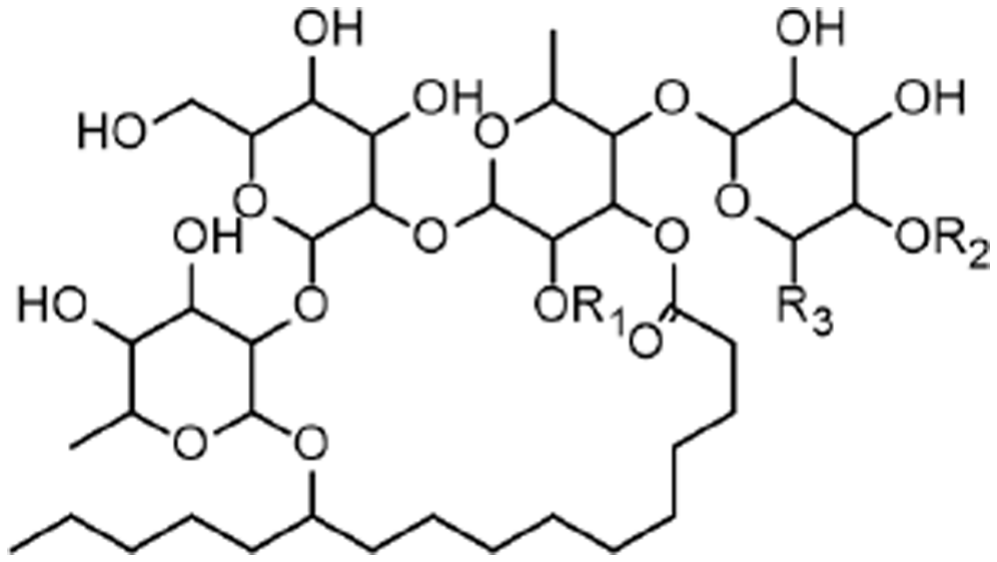								
No	RT (min)	m/z	Formula	Adducts	Compound	R_1_	R2	R_3_
RG-11	0.77	1035.5307	C_50_H_84_O_22_	M−H	Scammonin VIII	2-mba	Aga	CH_2_OH
RG-12	0.86	1019.5388	C_50_H_84_O_21_	M−H	Scammonin I/Scammonin VII	2-mba	Aga	CH_3_
RG-15	0.78	1017.521	C_68_H_120_O_24_	M−H	Scammonin IV	Aga	Aga	CH_3_

*Ac, acetic; Pa, propionic; Iba, isobutyric; 2-mba, 2R-methylbutyric; Aga, angelic acid; Octa, n-octanoic; Deca, n-decanoic; Dodeca, n-dodecanoic acid; Cna, trans-cinnamic acid; Glu, Glycosyl; Rha, Rhamnosyl. The asterisk (*) used in the table indicates explanatory notes for functional group abbreviations in table.

**Figure 3:**
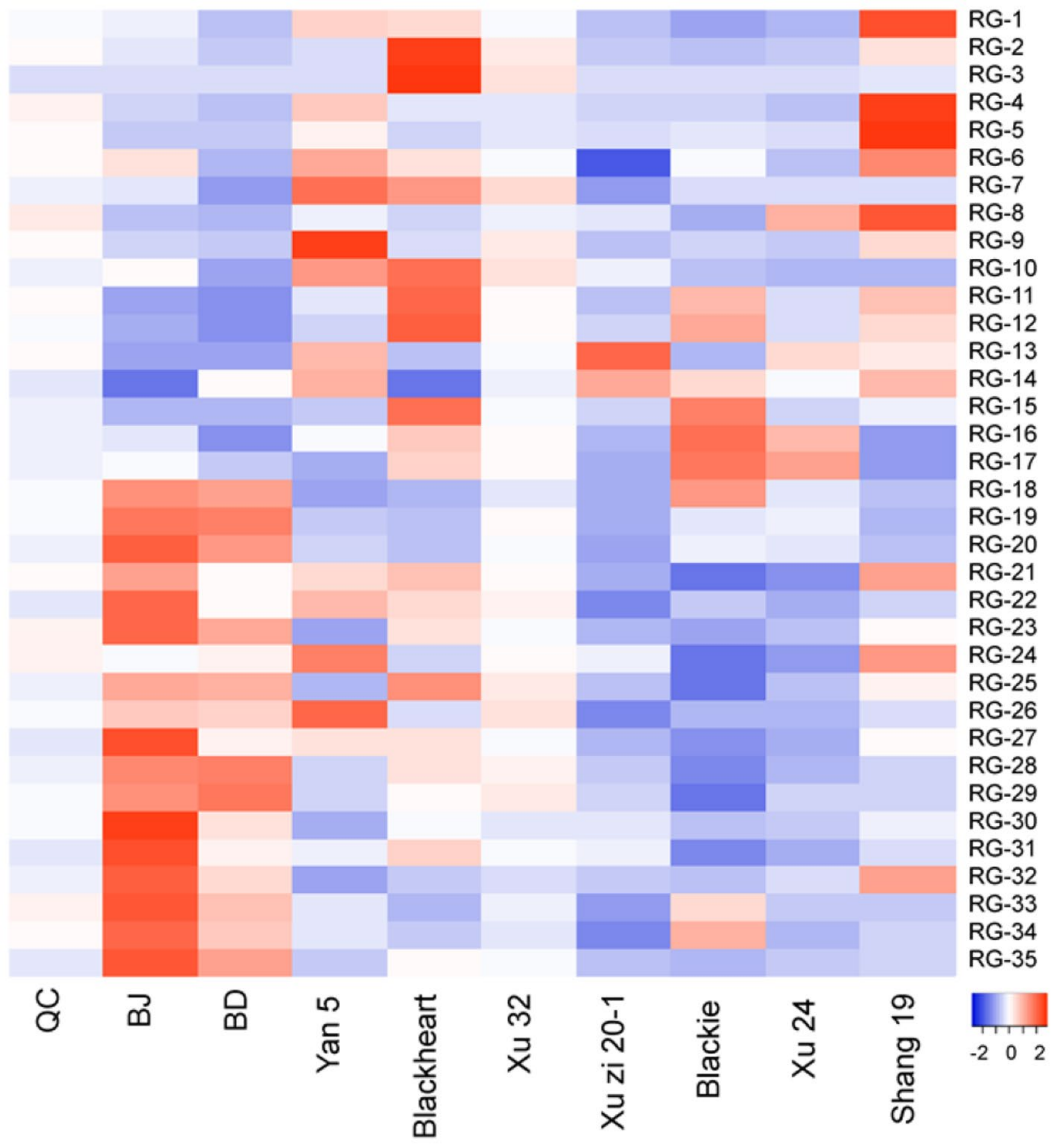
Heat map representations for the relative contents of significantly discriminant resin glycosides in nine sweet potato leaves. Metabolites were selected by variable importance in the projection (VIP) value > 1.0, *p*-value < 0.05.

The variations in key RGs were further analyzed and visualized through a heatmap with their relative abundance, as shown in Figure 3. Among them, Xu32 exhibited a neutral relative abundance of each RG type, closely aligning with those observed in the QC samples. Yan5 was characterized by higher abundances of cairicoside IV and its isomers (cairicoside A/ cairicoside B), as well as batatoside B and its isomers (batatotinoside I/ batatoside C/ batatoside D/ batatoside E/ batatoside F), which are all pentasaccharides featuring three acyl groups, including Cna and 2-mba, along with a saturated fatty acid side chain, either Deca or Dodeca. Blackheart exhibited a relative abundance of scammonins, including scammonin IV, scammonin VIII and scammonin I/ scammonin VII, which are tetra-saccharide. In these compounds, the macrolactonization takes place at C-3 of third sugar units, differing from other key RGs, where it typically occurs at the second sugar units. Xu Zi 20–1 predominantly displayed lower relative abundances (blue coloration) for most compounds. However, it exhibited significantly higher levels in intrapilosin VII and murucoidin IX/ murucoidin II compared to other samples. Differently, Blackie was rich in murucoidin XVIII/stoloniferin VIII and Murucoidin IV, both of which are pentasaccharide. Murucoidin XVIII is acylated with two groups: 2-mba and Octa, while Murucoidin IV is acylated with two 2-mba groups. A similar trend was observed in Xu24, which aligns with their close proximity in the PLS-DA plot. Shang19 contains a relatively high amount of batatoside B, and their isomers, which is similar to Yan5. The difference lies in the fact that it also has higher level of operculins (operculin IX/operculin X, operculin VII/operculin VIII), batatoside I/ batatoside H and simonin IV. Distinct from the samples mentioned above, the BJ sample exhibits a high relative abundance in over half of the RGs, indicating a distinct RG composition compared to other samples, which aligns with the PCA results. Similarly, the BD sample shows a comparable trend to BJ, as they are the same variety grown under different conditions.

Differences within the same class of secondary metabolites among cultivars are common in mother nature. For example, Phahlane et al. (9) reported that phenolic profiles in sweet potato leaves varied substantially among South African cultivars (“Ndou,” “Bophelo,” “Monate,” and “Blesbok”), the US cultivar “Beauregard,” and the Peruvian cultivar “199062.1.” Similarly, variation was observed in seed polyphenol accumulation among different faba bean cultivars, where multi-environment field trials indicated that genetic factors exert predominant control, with environmental influences acting as ancillary yet significant modulators (10). For RGs, studies explicitly addressing cultivar-level variation are still limited. Nevertheless, Kruse et al. (11) reported that substantial variability in the number and diversity of RGs occurs not only at the species level but also at the variety/accession level. In *Convolvulus arvensis*, accessions collected from two geographically distinct populations, Yakima (Washington) and Huntley (Montana), yielded 150 and 215 RGs, respectively, thereby indicating the critical roles of genotype, environment, and their interaction in shaping RG profiles (11).

### 3.3 Difference of RGs among Beniazuma and other cultivars

**Figure 4:**
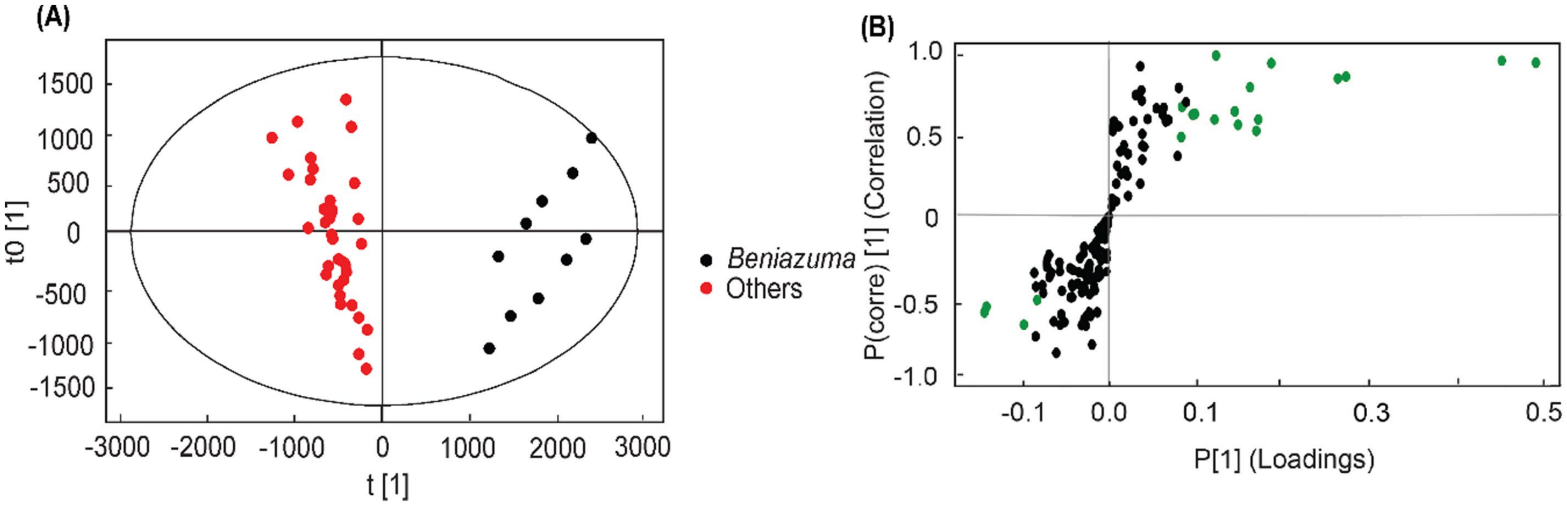
OPLS-DA on the UPLC-HRMS profiles of Group A (*Beniazuma* cultivars) vs. Group B (other cultivars): **(A)** Score plot; **(B)** S-plot. Green dots represent resin glycosides (RGs) with variable importance in projection (VIP) values > 1 and *p* < 0.05, which were considered the most discriminant metabolites.

To identify the RGs that significantly affected the separation of Beniazuma samples (BD and BJ) and other samples, OPLS-DA modeling was performed on the profiling data sets. A pronounced separation was observed in the score plot between two Beniazuma samples and remaining samples (Figure 4A). The OPLS-DA model explained more than 90% (R^2^Y) and predicted more than 88% (Q^2^) of the total variance. The S-plot was established by plotting covariance (p) against correlation (p-corr), and the potential biomarkers for discerning *Beniazuma* from other cultivars were achieved through filtering variables with VIP > 1 and *p* < 0.05 in the statistical analysis. In the S-plot, each scattered point represented a variable, with points further from the origin indicating greater contributions to the group differentiation. VIP is a weighted sum of squares derived from partial least square (PLS) weight and a value greater than one is generally utilized as a criterion to differentiate the important variables in the model. A total of 18 potential biomarkers were identified from the OPLS-DA (Figure 4B; Supplementary Table S3). Among them, the Multifidin I/ Quamoclin III and Cairicoside E are two strong contributors, which were four times higher in two Beniazuma samples than the rest of other cultivars, that can be used as biomarkers for distinguishing leaves from the Beniazuma variety.

### 3.4 Impact of growing conditions on RG profiles of two Beniazuma cultivars

**Figure 5:**
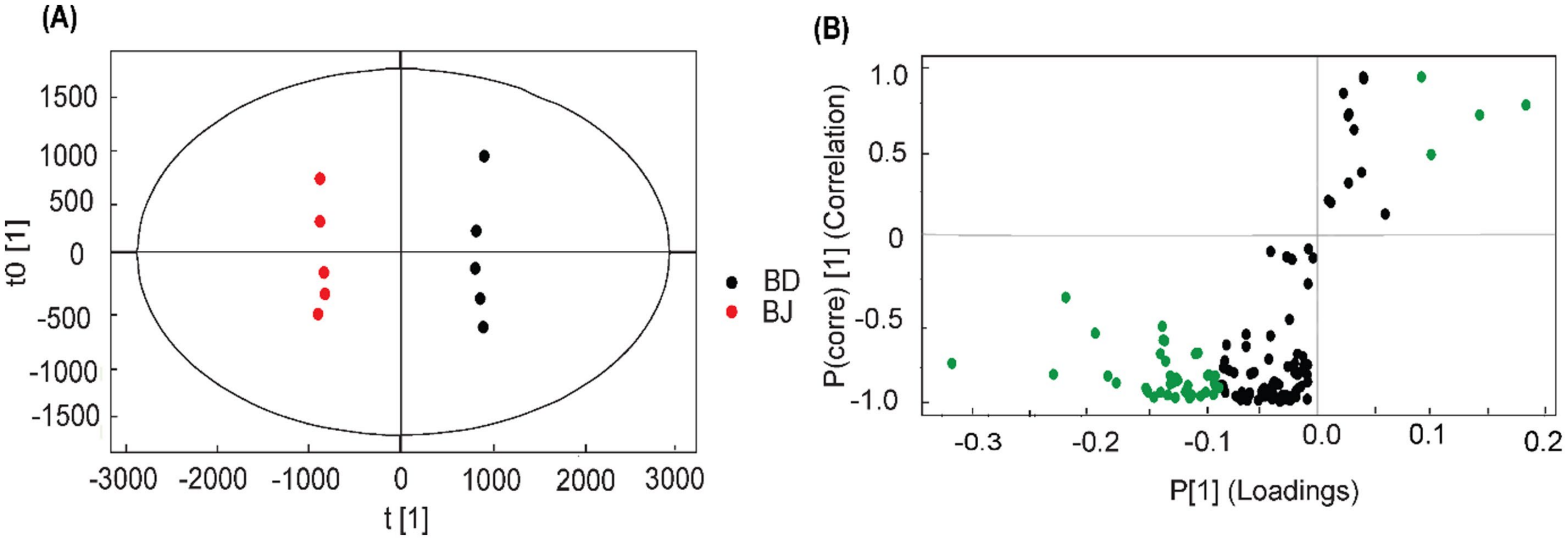
OPLS-DA of the two *Beniazuma* cultivars: **(A)** Score plot; **(B)** S-plot. Green dots represent resin glycosides (RGs) with variable importance in projection (VIP) values > 1 and *p* < 0.05, which were considered the most discriminant metabolites.

In this study, the two Beniazuma samples were grown under different conditions, BD was cultivated in a controlled hydroponic system, while BJ was grown using traditional soil-based system. The RG profiles of two Beniazuma samples were investigated and compared. In the PCA (Figure 1), both samples appeared in the lower right section of the score plot and were clustered together, but they could be further clearly categorized into two sub-groups with BDs are located further toward the bottom. Our results indicated that cultivation conditions significantly affected RGs profiles, even within the same variety. To identify the differential RGs, a similar method as mentioned above was applied to generate a new OPLS-DA model. The OPLS-DA model achieved a significant separation between the two groups and showed goodness-of-fit and high prediction ability (R^2^Y = 99%, Q^2^ = 98%) (Figure 5A). In total, 38 metabolites were filtered and selected with VIP value > 1, which were potential RGs responsible for the discrimination between the two cultivars (Figure 5B; Supplementary Table S4). Among them, there are four markers, murucoidin IX/ murucoidin II, batatinoside V/ pescaperin VI/ pescaperin VII, batatoside M, scammonin V, showed higher contents in hydroponically grown samples compared to those cultivated in soil. Pérez-Sanvicente et al. (12) found that variables such as temperature, soil texture, canopy cover, pH, and K^+^ concentration are strongly associated with the diversity of RGs in latex. These findings underscore the pivotal role of environmental factors, in addition to genetic background, in defining RGs expression.

### 3.5 Pancreatic lipase inhibition effects of SPL extracts

**Figure 6:**
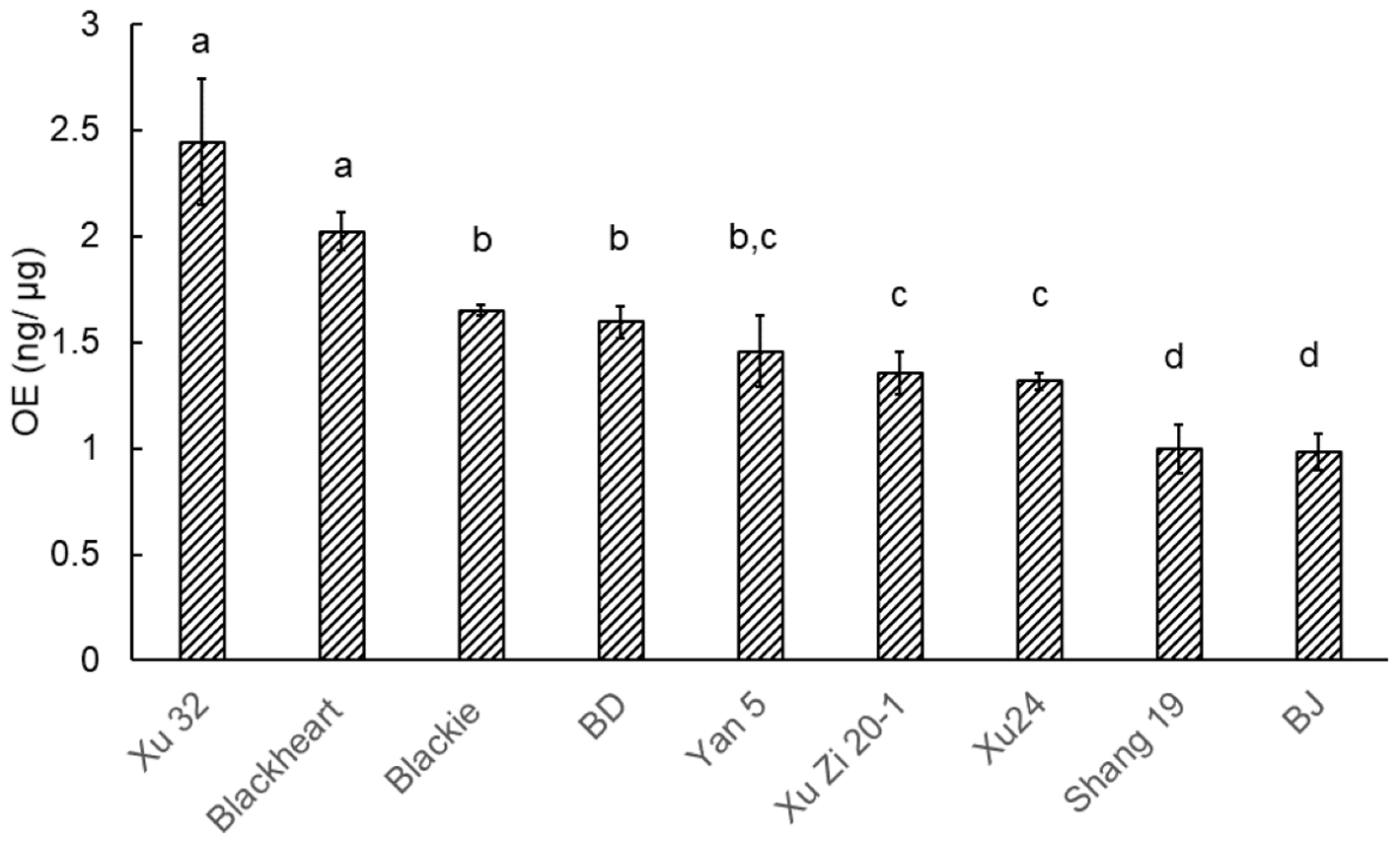
Pancreatic lipase inhibition activity of RG fractions from different cultivars of sweet potato leaves (Different lowercase letters indicated a significant difference at *p* < 0.05).

In a previous study, RGs have been demonstrated as lipase inhibitors, which are potential anti-obesity agents (3). In this study, a pNPP assay was used to evaluate the PL inhibitory capacity, with representative inhibition kinetics and dose–response curves of the SPL extracts provided in Supplementary Figure S3. To facilitate direct comparison, the PL inhibitory activities of each sample were summarized as OE values in Figure 6, where higher values indicate stronger inhibition. Among all samples, Xu32 and blackheart showed the highest inhibitory activity among all samples with OE value > 2 ng orlistat/ μg extracts, while BJ and Shang19 exhibited similar OE values of 1.00 ± 0.11 and 0.98 ± 0.07 ng orlistat/ μg extracts, respectively, which were significantly lower than the other samples. The substantial differences observed among cultivars may reflect genetic divergence, which influences RG biosynthetic patterns and, in turn, modulates PL inhibitory potential. Interestingly, the BD group exhibited higher PL inhibition than the BJ group despite sharing the same genetic background, indicating that growing conditions can further modulate biological activity within a single variety. These findings suggested cultivar selection as an essential initial step for achieving SPL with strong PL inhibition, with the top-performing genotype offering the most suitable framework for subsequent environmental optimization. Future research should focus on evaluating the effects of different biotic and abiotic factors on RG production, as the present study provides only preliminary insights based on two cultivation systems. Broadening the range of environmental conditions evaluated will be crucial to elucidate their impact on RG profiles and to decide the optimal parameters for maximizing PL inhibition.

### 3.6 Mode of action and structure–activity relationship of RGs and their lipase inhibition activity

In order to determine the potential key players, two OPLS-DA analyses were conducted to compare the RGs profile of Xu32 and Blackheart with others, respectively. Both models achieved significant separation between groups and showed goodness-of-fit and high prediction ability with a R^2^Y = 95%, Q^2^ = 93%; R^2^Y = 93%, Q^2^ = 89% for Xu32 and Blackheart, respectively (Supplementary Figure S4). According to the S-plot, 25 and 24 RGs were identified as key components (VIP > 1) that were responsible for the separation of Xu32 and blackhearts with other cultivars, respectively (Supplementary Tables S5, S6). Four RGs with retention times (RT) of 2.72 min, 9.28 min, 9.74 min, 9.87 min, appeared in both VIP tables.

The first compound (RT 2.72 min) showed [M+COOH]^−^ at *m/z* 1441.7764 in negative ESI mode, and the calculated molecular formula C_72_H_116_O_26_ was fitted based on the analysis of its elemental composition and fractional isotope abundance. The fragment ion peaks at *m/z* 1395.7701[M−H]^−^, 1213.6011[M-182(C_12_H_22_O)]^−^, 1127.5358 [1213–84(C_5_H_8_O)]^−^, 545.3287 [1129–162 (C_6_H_10_O_5_)-130 (C_9_H_6_O)-2 ×146 (C_6_H_10_O_4_)]^−^, 431.2264 [545–113 (C_6_H_8_O_2_)]^−^ were detected and analyzed. Thus, this peak was tentatively assigned as batatoside M.

The second compound (RT 9.28 min), has molecular ion at m/z 1311.7347 [M+COOH]^−^ in negative ESI mode. It fragmented into daughter ions at *m/z*1265.7283 [M−H]^−^, 1181.6669 [M−H− 84(C_5_H_8_O)]^−^, 1083.5781 [M−H−182 (C_12_H_22_O)]^−^, 837.4576[1083–162 (C_6_H_10_O_5_) - 84 (C_5_H_8_O)]^−^, 545.3315 [837–2 × 146 (C_6_H_10_O_4_) -84(C_5_H_8_O)]^−^, 417.2854 and this fits nicely with cairicoside F or its isomers (murucoidin VI/ murucoidin XI/ quamoclin IV).

The third compound (RT 9.74 min) shows a molecular ion peak m/z 1295.7391 [M+COOH]^−^ (C_63_H_110_O_24_)and fragments into m/z 1249.7336 [M−H]^−^, 1165.6742 [M−H – 84(C_5_H_8_O)]^−^, 1067.5668 [M−H – 182 (C_12_H_22_O)]^−^, 921.5064 [1067–146 (C_6_H_10_O_4_)] ^-^, 545.3331 [921–2 × 146 (C_6_H_10_O_4_) – 84 (C_5_H_8_O)]^−^, 417.2858, which fits to the patterns of batatinoside VIII/ pescaprein III/ stoloniferin X.

The forth compound (RT 9.87 min) has molecular ion at m/z 1311.7349 was tentatively assigned as simonin II, with the fragment ion peaks at 1265.7296 [M−H]^−^, 1163.6582 [M−H− 84 (C_5_H_8_O)-18 (H_2_O)]^−^, 832.6166 [M−H− 433]^−^, 670.5994 [832–162 (C_6_H_10_O_5_)]^−^, 433.2806 [162 (C_6_H_10_O_5_) + 272(C_16_ H_32_ O_3_)-H]^−^ were detected.

**Figure 7:**
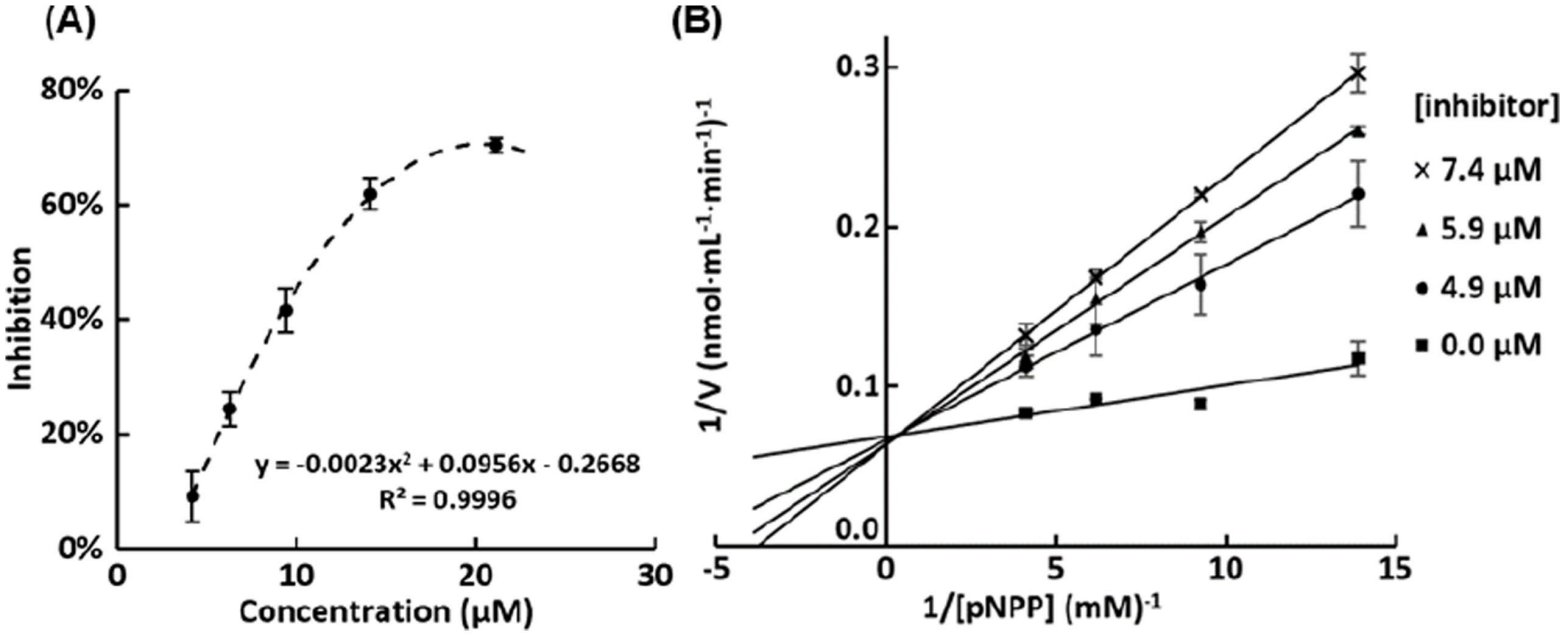
**(A)** Representative dose-response curves for the inhibition of pancreatic lipase by pescaprein III. **(B)** Lineweaver–Burk plot for pNPP hydrolysis by pancreatic lipase in the presence of different concentrations of pescaprein III.

Overall, all these compounds are pentasaccharides, featuring 2-mba and Dodeca as side chains. To further elucidate the structure–activity relationship, pescaprein III (the third compound) was selected for mechanism study. Pescaprein III was purified by reversed-phase HPLC on a C18 column using methanol and water as mobile phase, and its structure was confirmed by NMR (Supplementary Figure S5). As shown in Figure 7A, pescaprein III exhibited PL inhibitory activity with an IC_50_ of 11.0 ± 0.7 μM. The Lineweaver–Burk plot (Figure 7B) revealed a competitive mode of inhibition, as the slopes increased with increasing concentrations of pescaprein III, whereas the y-intercepts remained nearly unchanged. Without addition of pescaprein III, V_max_ of PL was 14.6 μM/min, and K_m_ was 47 μM. K_i_ (enzyme inhibition constant) of the pescaprein III was also calculated to be 1.70 ± 0.17 μM.

To substantiate the active moiety, the pescaprein III was subjected to alkaline hydrolysis. The resulting product, simonic acid B, was obtained by solvent extraction and HPLC separation (Mass spectrum shown in Supplementary Figure S6) (13). With the ester bonds hydrolyzed, simonic acid B had significantly lower PL inhibition (IC_50_ = 400 μM) compared with pescaprein III. This indicates that the long chain fatty acid esters or the macrocyclic lactone on resin glycoside molecule may contribute to the competitive inhibition property, since PL recognizes long carbon chains specifically for pNPP hydrolysis. Similarly, amphiphilic saponins are well-known lipase inhibitors, and they also showed competitive inhibition mechanism against lipase (14, 15). In fact, orlistat, the clinically approved lipase inhibitor, shares similar structural properties with resin glycoside. The lactone structure of orlistat was reported as the crucial active moiety since it bound covalently to Ser152 in PL active site (16). Our results highlighted that long acyl side chains and lactone structure, rather than the oligosaccharide core, govern the inhibitory activity, further studies are needed to delineate the specific contribution of acyl chain length, as such insights are critical for a comprehensive understanding of structure–activity relationships of RGs and for predicting their inhibitory potential.

To date, research on RGs in sweet potatoes remains limited to isolation and structural characterizations. The first known study was conducted in Japan in 1992 by Noda et al., using the roots of a locally grown Brazilian cultivar (cv. Simon) (17). Five new ether-soluble RGs, simonins I-V, have been identified. Notably, simonin I is the first reported RG to feature an aromatic acid, specifically trans-cinnamic acid, as a substituent. The scholarly pursuits in this field exhibit an apparent hiatus until Escalante-Sánchez and colleagues isolated batatinosides I–VI from roots of sweet potato, as well as batatins I and II, both of which are dimers derived from batatinoside I (18, 19). Meanwhile, Noda et al. reported the isolation of four RGs from *Ipomoea batatas* L. LAM., Kokei No. 14, which are monomeric containing similar glycosidic and organic acids to those of simonins and batatinosides (20). A further study of these compounds revealed their activity in reversal of multidrug resistance in vinblastine resistant human breast carcinoma cells (MCF-7/Vin) by batatinoside IV and batatins I − II (21). More recently, Yin et al. isolated 21 types of new resin glycosides from sweet potato, including batataosides I-V as well as batatosides A-P (22–25). Among them, batatoside E, batatoside L and batatoside O proved to be mild inhibitors in the proliferation of Hep-2 cells (23, 25). In 2010, a research group from Japan identified ipomotatoside A-D (26). Interestingly, Ipomotatoside A exhibited promising anti-inflammatory activity, with potency against both COX-1 and COX-2 comparable to that of aspirin. In 2011, the second batch of RG dimers, batatins III–VI, which represent the first examples of ester-type dimers consisting of two units of the hetero-tetrasaccharide operculinic acid C, were isolated from a white-skin variety in Mexico by Rosas-Ramírez and colleagues (27). In the next 4 years, the same group isolated another five dimers batatins VII-XI and three monomers batatinosides VII − IX, from sweet potato (28, 29). After 3 years, Ono and colleagues isolated four new RGs, murasakimasarins I–IV, from the tubers of *Ipomoea batatas* (L.) Lam. (‘Murasakimasari’). Murasakimasarin III is the first representative of RGs with 10-methylundecanoic acid as the organic acid ester (30). From these studies and ours, the most salient feature of the RGs in morning glory family vegetables is their complex structural variations due to the isomeric form lactone, number of sugars (4 and 5), acid moieties in two ester substituent sides. The structural variations would impact their biological activity. To tap on their potential for human health promotion as active ingredients, it is only practical to treat the RGs from sweet potato leaves as a mixture by fractionation and enrichment instead of isolation of single compound. To this end, metabolomic approach using UHPLC-Q-TOF-MS is a method of choice to characterize the extracts from different sources of sweet potatoes. Correlation of the RG profiles and the bioactivity relationship is important to guide the selection of the cultivars for commercial exploitation of RGs. Further study should extend current research beyond *in vitro* assays to animal models to provide deeper insights and clarify the underlying mechanisms, which are essential for harnessing these RGs in the development of functional foods.

## 4 Conclusion

In summary, UHPLC-Q-TOF-MS/MS is a powerful method for identifying RGs in SPL extracts. The PCA analysis facilitates an objective evaluation of the obtained MS data, demonstrating the differences among specific cultivars. Additionally, the lipase inhibitory activity of nine different cultivars was evaluated, revealing the notable influence of both cultivar and growing conditions. The leaves, despite originating from same cultivar, exhibited varying activities under different growing conditions. Among the samples, Xu32 and blackheart showed the highest activity. Comparative analysis suggested that compounds such as batatoside M, cairicoside F/ murucoidin VI/ murucoidin XI/ quamoclin IV, simon II, batatinoside VIII/ pescaprein III/ stoloniferin X may be the predominant contributors to the lipase inhibitory activity in the SPLs. There are close to 2,000 varieties of sweet potato and our current work only serves as first step for build a greater database of RGs by expanding the sample sizes from over 1800 cultivars collected in Xuzhou Sweet Potato Institute, which serves a sample bank for precision agriceuticals in human health promotion.

## Data Availability

The original contributions presented in the study are included in the article/Supplementary material, further inquiries can be directed to the corresponding author/s.

## Footnotes

1
http://scifinder.cas.org
