# Supplementary material for: UHPLC-Q-TOF-MS/MS profiling of resin glycosides and their lipase inhibitory activity in leaves of selected sweet potato (Ipomoea batatas L.) cultivars
Source: Front Nutr. 2026 Mar 19;13:1783378. doi: 10.3389/fnut.2026.1783378 (PMC13073091; doi:10.3389/fnut.2026.1783378)
Supplement: Supplementary file 1 [file supplementary_file_1.docx]

**UHPLC -Q-TOF-MS/MS profiling of resin glycosides and their lipase inhibitory activity in leaves of selected sweet potato (*Ipomoea batatas* L.) cultivars**

Yi Lin^a,*^, Zhixuan Song^a^, Qingtong Xie^a^, Yuyun Lu^a^, Molan Zhang^a^,

Joanne Yi Hui Toy^a^, Dejian Huang^a,b,*^

*^a^Department of Food Science and Technology, National University of Singapore, 2 Science Drive 2, Singapore 117542*

*^b^National University of Singapore (Suzhou) Research Institute, 377 Linquan Street, Suzhou, Jiangsu, 215123, China*

Supporting information

*Corresponding authors:

Dejian Huang, dejian@nus.edu.sg, 2 Science Drive 2, Singapore 117542, tel. +(65) 6516 8821

Yi Lin, [liny@nus.edu.sg](mailto:liny@nus.edu.sg), 2 Science Drive 2, Singapore 117542, tel. +(65) 6516 1656

**Table S1.** List of RG in self-constructed library.

| **No** | **Compound** | **No** | **Compound** | **No** | **Compound** | **No** | **Compound** | **No** | **Compound** | **No** | **Compound** |
| --- | --- | --- | --- | --- | --- | --- | --- | --- | --- | --- | --- |
| 1 | Batataoside I | 32 | Batatoside G | 63 | Impotatoside D | 94 | Murasakimasarin II | 125 | Operculin II | 156 | Orizabin XVI |
| 2 | Batataoside II | 33 | Batatoside H | 64 | Leptophyllin A | 95 | Murasakimasarin III | 126 | Operculin IX | 157 | Orizabin XVII |
| 3 | Batataoside III | 34 | Batatoside I | 65 | Leptophyllin B | 96 | Murasakimasarin IV | 127 | Operculin V | 158 | Orizabin XVIII |
| 4 | Batataoside IV | 35 | Batatoside J | 66 | Mammoside A | 97 | Muricatin I | 128 | Operculin VI | 159 | Orizabin XX |
| 5 | Batataoside V | 36 | Batatoside K | 67 | Mammoside B | 98 | Muricatin II | 129 | Operculin VII | 160 | Orizabin XXI |
| 6 | Batatin I | 37 | Batatoside L | 68 | Mammoside H1 | 99 | Muricatin III | 130 | Operculin VIII | 161 | Pescaprein I |
| 7 | Batatin II | 38 | Batatoside M | 69 | Mammoside H2 | 100 | Muricatin IV | 131 | Operculin X | 162 | Pescaprein II |
| 8 | Batatin III | 39 | Batatoside N | 70 | Marubajalapin I | 101 | Muricatin V | 132 | Operculin XI | 163 | Pescaprein III |
| 9 | Batatin IV | 40 | Batatoside O | 71 | Marubajalapin II | 102 | Muricatin VI | 133 | Operculin XII | 164 | Pescaprein IV |
| 10 | Batatin V | 41 | Batatoside P | 72 | Marubajalapin III | 103 | Muricatin VII | 134 | Operculin XIII | 165 | Pescaprein IX |
| 11 | Batatin VI | 42 | Cairicoside A | 73 | Marubajalapin IV | 104 | Murucoidin I | 135 | Operculin XIV | 166 | Pescaprein V |
| 12 | Batatin VII | 43 | Cairicoside B | 74 | Marubajalapin IX | 105 | Murucoidin II | 136 | Operculin XV | 167 | Pescaprein VI |
| 13 | Batatin VIII | 44 | Cairicoside C | 75 | Marubajalapin V | 106 | Murucoidin III | 137 | Operculin XVI | 168 | Pescaprein VII |
| 14 | Batatin IX | 45 | Cairicoside D | 76 | Marubajalapin VI | 107 | Murucoidin IV | 138 | Operculin XVII | 169 | Pescaprein VIII |
| 15 | Batatin X | 46 | Cairicoside E | 77 | Marubajalapin VII | 108 | Murucoidin IX | 139 | Operculin XVIII | 170 | Pescaprein X |
| 16 | Batatin XI | 47 | Cairicoside F | 78 | Marubajalapin VIII | 109 | Murucoidin V | 140 | Orizabin I | 171 | Pescaprein XI |
| 17 | Batatinoside I | 48 | Cairicoside I | 79 | Marubajalapin X | 110 | Murucoidin VI | 141 | Orizabin II | 172 | Pescaprein XII |
| 18 | Batatinoside II | 49 | Cairicoside II | 80 | Marubajalapin XI | 111 | Murucoidin VII | 142 | Orizabin III | 173 | Pescaprein XIII |
| 19 | Batatinoside III | 50 | Cairicoside III | 81 | Merremoside a | 112 | Murucoidin VIII | 143 | Orizabin IV | 174 | Pescaprein XIV |
| 20 | Batatinoside IV | 51 | Cairicoside IV | 82 | Merremoside b | 113 | Murucoidin X | 144 | Orizabin IX | 175 | Pescaprein XIX |
| 21 | Batatinoside IX | 52 | Digitatajalapin I | 83 | Merremoside c | 114 | Murucoidin XI | 145 | Orizabin V | 176 | Pescaprein XV |
| 22 | Batatinoside V | 53 | Intrapilosin I | 84 | Merremoside d | 115 | Murucoidin XII | 146 | Orizabin VI | 177 | Pescaprein XVI |
| 23 | Batatinoside VI | 54 | Intrapilosin II | 85 | Merremoside e | 116 | Murucoidin XIII | 147 | Orizabin VII | 178 | Pescaprein XVII |
| 24 | Batatinoside VII | 55 | Intrapilosin III | 86 | Merremoside f | 117 | Murucoidin XIV | 148 | Orizabin VIII | 179 | Pescaprein XVIII |
| 25 | Batatinoside VIII | 56 | Intrapilosin IV | 87 | Merremoside g | 118 | Murucoidin XIX | 149 | Orizabin X | 180 | Pescaprein XX |
| 26 | Batatoside A | 57 | Intrapilosin V | 88 | Merremoside h1 | 119 | Murucoidin XV | 150 | Orizabin XI | 181 | Pescaprein XXI |
| 27 | Batatoside B | 58 | Intrapilosin VI | 89 | Merremoside h2 | 120 | Murucoidin XVI | 151 | Orizabin XII | 182 | Pescaprein XXII |
| 28 | Batatoside C | 59 | Intrapilosin VII | 90 | Merremoside i | 121 | Murucoidin XVII | 152 | Orizabin XIII | 183 | Pescaprein XXIII |
| 29 | Batatoside D | 60 | Impotatoside A | 91 | Multifidin I | 122 | Murucoidin XVIII | 153 | Orizabin XIV | 184 | Pescaprein XXIV |
| 30 | Batatoside E | 61 | Impotatoside B | 92 | Multifidin II | 123 | Murucoidin XX | 154 | Orizabin XIX | 185 | Pescaprein XXIX |
| 31 | Batatoside F | 62 | Impotatoside C | 93 | Murasakimasarin I | 124 | Operculin I | 155 | Orizabin XV | 186 | Pescaprein XXV |

**Table S1.** Continued.

| **No** | **Compound** | **No** | **Compound** | **No** | **Compound** | **No** | **Compound** | **No** | **Compound** | **No** | **Compound** |
| --- | --- | --- | --- | --- | --- | --- | --- | --- | --- | --- | --- |
| 187 | Pescaprein XXVI | 197 | Quamoclin V | 207 | Scammonin VIII | 217 | Stoloniferin IX | 227 | Tricolorin B | 237 | Tuguajalapin V |
| 188 | Pescaprein XXVII | 198 | Quamoclin VI | 208 | Simonin I | 218 | Stoloniferin V | 228 | Tricolorin C | 238 | Tuguajalapin VI |
| 189 | Pescaprein XXVIII | 199 | Quamoclin VII | 209 | Simonin II | 219 | Stoloniferin VI | 229 | Tricolorin D | 239 | Tuguajalapin VII |
| 190 | Pescaprein XXX | 200 | Scammonin I | 210 | Simonin III | 220 | Stoloniferin VI | 230 | Tricolorin E | 240 | Tuguajalapin VIII |
| 191 | Pescaproside A | 201 | Scammonin II | 211 | Simonin IV | 221 | Stoloniferin VII | 231 | Tricolorin F | 241 | Tuguajalapin X |
| 192 | Pescaproside B | 202 | Scammonin III | 212 | Simonin V | 222 | Stoloniferin VIII | 232 | Tuguajalapin I | 242 | Tuguajalapin XI |
| 193 | Quamoclin I | 203 | Scammonin IV | 213 | Stoloniferin I | 223 | Stoloniferin X | 233 | Tuguajalapin II | 243 | Woodrosin I |
| 194 | Quamoclin II | 204 | Scammonin V | 214 | Stoloniferin II | 224 | Stoloniferin XI | 234 | Tuguajalapin III | 244 | Woodrosin II |
| 195 | Quamoclin III | 205 | Scammonin VI | 215 | Stoloniferin III | 225 | Stoloniferin XII | 235 | Tuguajalapin IV |  |  |
| 196 | Quamoclin IV | 206 | Scammonin VII | 216 | Stoloniferin IV | 226 | Tricolorin A | 236 | Tuguajalapin IX |  |  |

**Table S2.** Identification of RG in sweet potato leaves

| **No** | **RT**  **(min)** | **m/z** | **Formula** | **Adducts** | **Mass error**  **（ppm）** | **Proposed compounds** |
| --- | --- | --- | --- | --- | --- | --- |
| **1** | 0.67 | 1113.5646 | C51H88O23 | M+FA-H | -4.9319 | Batataoside I/ Murucoidin I |
| **2** | 0.72 | 1053.5565 | C49H84O21 | M+FA-H | 7.7185 | Tricolorin B |
| **3** | 0.73 | 1051.5577 | C50H86O20 | M+FA-H | -11.6882 | Mammoside A/Merremoside A/ Muticatin I/ Murucoidin XV |
| **4** | 0.76 | 753.385 | C34H60O15 | M+FA-H | -9.0433 | Tricolorin F |
| **5** | 0.77 | 1035.5307 | C50H84O22 | M-H | -7.1967 | Scammonin VIII |
| **6** | 0.78 | 1017.521 | C50H82O21 | M-H | -6.4653 | Scammonin IV |
| **7** | 0.86 | 1019.5388 | C50H84O21 | M-H | -4.3458 | Scammonin I/Scammonin VII |
| **8** | 0.95 | 1179.6854 | C58H102O21 | M+FA-H | -3.63820 | Marubajalapin V/ Marubajalapin IV/ Marubajalapin III |
| **9** | 1.06 | 1153.5938 | C55H94O25 | M-H | -6.3791 | Mammoside H1 |
| **10** | 1.2 | 1183.6366 | C56H98O23 | M+FA-H | -10.1264 | Pescaprein VI/ Batainoside V/Pescaprein VII |
| **11** | 1.22 | 937.5045 | C45H78O20 | M-H | 3.3346 | Scammonin II |
| **12** | 1.25 | 1269.6871 | C60H104O25 | M+FA-H | 1.7808 | Murucoidin XIX |
| **13** | 1.27 | 1429.7306 | C66H112O30 | M+FA-H | 6.1604 | Woodrosin I |
| **14** | 1.28 | 1311.7286 | C63H110O25 | M+FA-H | -2.5054 | Murucoidin VI/Cairicoside F |
| **15** | 1.33 | 901.4728 | C40H72O19 | M+FA-H | 9.1444 | Merremoside I |
| **16** | 1.39 | 1133.6836 | C58H102O21 | M-H | -0.4545 | Marubajalapin III/ Marubajalapin IV/ Marubajalapin V |
| **17** | 1.64 | 1295.7342 | C63H110O24 | M+FA-H | -2.134 | Stoloniferin X |
| **18** | 1.85 | 1149.6749 | C57H100O20 | M+FA-H | -3.7524 | Pescaprein V/ Murucoidin XIV |
| **19** | 1.85 | 1441.7760 | C72H116O26 | M+FA-H | 1.6747 | Intrapilosin V/ Intrapilosin IV/ Intrapilosin VI/ |
| **20** | 1.86 | 1265.7273 | C63H110O25 | M-H | 0.7501 | Murucoidin XI / Quamoclin IV |
| **21** | 1.87 | 1181.6712 | C58H102O24 | M-H | 2.0046 | Batatinoside VII/Batatinoside IX/ Operculin XIII/ |
| **22** | 2.01 | 1133.5554 | C50H88O25 | M-H, M+FA-H | -3.9053 | Quamoclin VII |
| **23** | 2.03 | 1277.7584 | C64H112O22 | M+FA-H | -3.5483 | Marubajalapin VIII |
| **24** | 2.18 | 1379.7734 | C72H116O25 | M-H | 0.0414 | Batatinoside I/ Pescaprein XXI/ Pescaprein XXII |
| **25** | 2.23 | 1411.7651 | C71H114O25 | M+FA-H | 1.4198 | Batatoside O |
| **26** | 2.28 | 1213.6330 | C56H96O25 | M+FA-H | 9.1437 | Murucoidin VIII |
| **27** | 2.28 | 1137.6486 | C56H98O23 | M-H | 5.2532 | Pescaprein VI/Pescaprein VII/ Batatinoside V |
| **28** | 2.28 | 1341.6750 | C66H104O25 | M+FA-H | -7.6195 | Pescaprein XXIX |
| **29** | 2.36 | 1295.7170 | C66H106O22 | M+FA-H | 1.0081 | Batatoside K/ Batatoside L |
| **30** | 2.37 | 1425.7811 | C72H116O25 | M+FA-H | 1.6945 | Pescaprein XXI/Pescaprein XXII |
| **31** | 2.44 | 1183.6231 | C55H94O24 | M+FA-H | 9.9937 | Murucoidin IX/Murucoidin II |
| **32** | 2.45 | 1379.7725 | C72H116O25 | M-H | -0.5456 | Batatoside D/Batatotinoside I/ Batatoside E/ Batatoside F |

| **33** | 2.47 | 1425.7797 | C72H116O25 | M+FA-H | 0.6657 | Pescaprein XXII / Pescaprein XXI |
| --- | --- | --- | --- | --- | --- | --- |
| **34** | 2.61 | 1037.5866 | C50H88O19 | M+FA-H | -3.6222 | Batatinoside II |
| **35** | 2.62 | 1137.6394 | C56H98O23 | M-H | -2.8283 | Pescaprein VII/ Pescaprein VI/ batatinoside V |
| **36** | 2.62 | 1381.8043 | C68H120O25 | M-H, M+FA-H | -4.3468 | Operculin VII/ Operculin VIII |
| **37** | 2.64 | 1169.6245 | C56H98O25 | M-H | -6.7675 | Operculin XVII |
| **38** | 2.72 | 1441.7764 | C72H116O26 | M-H, M+FA-H | 1.9595 | Batatoside M |
| **39** | 2.78 | 1343.6780 | C65H102O26 | M+FA-H | 10.7066 | Murucoidin XVII/ Cairicoside C/ Cairicoside I |
| **40** | 2.91 | 1153.6310 | C56H98O24 | M-H | -5.6829 | Operculin XIV/ Batatinoside VI |
| **41** | 2.91 | 1267.6364 | C64H100O25 | M-H | -9.1886 | Batataoside I/ Batataoside II/ Batatoside A |
| **42** | 2.98 | 1197.6352 | C56H96O24 | M-H, M+FA-H | 6.7828 | Murucoidin III |
| **43** | 2.99 | 1397.8066 | C68H120O26 | M-H, M+FA-H | 1.2211 | Operculin IX/ Operculin X |
| **44** | 3.02 | 1051.5691 | C50H86O20 | M+FA-H | -0.3518 | Muricatin I/ Muricatin III |
| **45** | 3.11 | 1213.6270 | C56H96O25 | M+FA-H | 4.0427 | Murucoidin V/ Murucoidin IV |
| **46** | 3.3 | 1337.7982 | C66H116O24 | M+FA-H | 11.0762 | Simonin V |
| **47** | 3.35 | 1051.5732 | C50H86O20 | M+FA-H | 3.7261 | Murucoidin XV /Merremoside A/ Murucoidin XVI |
| **48** | 3.44 | 1327.6751 | C65H102O25 | M+FA-H | 4.5492 | Batataoside III /Pescaprein XXVII |
| **49** | 3.46 | 1343.6685 | C65H102O26 | M+FA-H | 3.3558 | Cairicoside I/ Cairicoside C/ Intrapilosin I/ Murucoidin XVII |
| **50** | 3.47 | 1351.7967 | C68H120O26 | M-H | -2.0673 | Operculin X |
| **51** | 3.55 | 1121.6485 | C55H96O20 | M+FA-H | 0.7895 | Stoloniferin XI/ Stoloniferin XII |
| **52** | 3.63 | 1213.6298 | C56H96O25 | M+FA-H | 6.4619 | Murucoidin IV |
| **53** | 3.88 | 1255.6727 | C59H102O25 | M-H, M+FA-H | 2.8574 | Quamoclin III/ Multifidin I |
| **54** | 4.03 | 1313.6641 | C64H100O25 | M+FA-H | 8.2965 | Batatoside A/ Batataoside I/ Batataoside II |
| **55** | 4.05 | 1483.8284 | C75H122O26 | M-H, M+FA-H | 5.3962 | Intrapilosin VII |
| **56** | 4.39 | 1343.6712 | C65H102O26 | M+FA-H | 5.4505 | Murucoidin XVII/ Cairicoside I |
| **57** | 4.68 | 1019.6168 | C52H92O19 | M-H | 0.7317 | Batatinoside III |
| **58** | 4.79 | 1343.6722 | C65H102O26 | M+FA-H | 6.2406 | Cairicoside C/ Cairicoside I |
| **59** | 4.87 | 1105.6522 | C56H98O21 | M-H | -0.4971 | Marubajalapin I/ Marubajalapin II |
| **60** | 4.88 | 1321.7788 | C69H112O21 | M+FA-H | 8.6323 | Simonin I |
| **61** | 5.12 | 1239.6773 | C59H102O24 | M-H, M+FA-H | 2.5242 | Murucoidin XVIII/ Stoloniferin VIII |
| **62** | 5.19 | 1343.6720 | C65H102O26 | M-H, M+FA-H | 6.0309 | Intrapilosin I/Cairicoside I/Cairicoside C/ Murucoidin XVII |
| **63** | 5.3 | 1247.7823 | C64H114O20 | M+FA-H | -5.2102 | Operculin XI /Operculin VI |
| **64** | 5.61 | 1083.5603 | C50H86O22 | M+FA-H | 0.9720 | Orizabin V |
| **65** | 5.61 | 1255.6722 | C59H102O25 | M-H, M+FA-H | 2.4693 | Multifidin I/ Quamoclin III |
| **66** | 5.62 | 1449.8468 | C77H126O25 | M-H | -3.2859 | Batataoside V |
| **67** | 5.73 | 1239.6762 | C59H102O24 | M-H, M+FA-H | 1.5471 | Stoloniferin VIII/ Murucoidin XVIII |
| **68** | 5.85 | 1365.7969 | C71H116O22 | M-H, M+FA-H | 2.1735 | Batatoside J |
| **69** | 6.26 | 1409.8394 | C70H124O25 | M-H, M+FA-H | -1.4688 | Operculin I |

| **70** | 6.35 | 1253.6932 | C60H104O24 | M+FA-H | 2.6768 | Stoloniferin II |
| --- | --- | --- | --- | --- | --- | --- |
| **71** | 6.38 | 1243.6719 | C58H102O25 | M-H, M+FA-H | 2.2669 | Operculin XVIII/ Operculin XVI |
| **72** | 7.02 | 1151.6664 | C56H98O21 | M+FA-H | 7.3582 | Marubajalapin II/ Marubajalapin I |
| **73** | 7.22 | 1409.8423 | C70H124O25 | M+FA-H | 0.6483 | Operculin V/ Operculin I |
| **74** | 7.53 | 1369.7216 | C68H108O25 | M+FA-H | 4.0669 | Pescaprein XXVIII |
| **75** | 7.56 | 1067.5612 | C51H88O23 | M-H, M+FA-H | -2.9712 | Murucoidin I/ Batataoside IV |
| **76** | 7.59 | 1269.6910 | C60H104O25 | M+FA-H | 4.9673 | Murucoidin XX |
| **77** | 7.8 | 1267.7093 | C61H106O24 | M-H, M+FA-H | 3.0459 | Pescaprein XXX/ Stoloniferin III/ Stoloniferin IX |
| **78** | 8.44 | 1207.7245 | C60H106O21 | M+FA-H | 3.1304 | Marubajalapin VI |
| **79** | 8.53 | 1385.7152 | C68H108O26 | M+FA-H | 3.0849 | Cairicoside D/ Cairicoside III/ Intrapilosin II |
| **80** | 8.54 | 1339.7108 | C68H108O26 | M-H | 3.8931 | Cairicoside III/Cairicoside D |
| **81** | 8.57 | 1283.7032 | C61H106O25 | M-H, M+FA-H | 2.2016 | Cairicoside E |
| **82** | 8.7 | 1267.7070 | C61H106O24 | M+FA-H | 1.1063 | Stoloniferin IX/ Pescaprein XXX/ Stoloniferin III |
| **83** | 8.89 | 1413.7463 | C70H112O26 | M+FA-H | 2.844 | Cairicoside A/ Cairicoside B/ Cairicoside IV |
| **84** | 8.95 | 1339.7210 | C68H108O26 | M-H | 11.5156 | Cairicoside III/ Cairicoside D |
| **85** | 8.96 | 1227.6796 | C58H102O24 | M+FA-H | 4.4650 | Batatinoside IX/ Batatinoside VII/ Operculin XIII |
| **86** | 9 | 1267.7009 | C61H106O24 | M+FA-H | -3.8177 | Stoloniferin III/ Pescaprein XXX/ Stoloniferin IX |
| **87** | 9 | 1263.7032 | C63H108O25 | M-H | -5.9204 | Digitatajalapin I |
| **88** | 9 | 1383.7384 | C69H110O25 | M+FA-H | 4.9171 | Batatoside G |
| **89** | 9 | 1383.7384 | C69H110O25 | M+FA-H | 4.9171 | Pescaprein XII |
| **90** | 9.01 | 1283.7044 | C61H106O25 | M-H, M+FA-H | 3.1123 | Leptophyllin B |
| **91** | 9.23 | 1211.6657 | C58H102O23 | M+FA-H | -11.7562 | Pescaprein I |
| **92** | 9.25 | 1283.7047 | C61H106O25 | M+FA-H | 3.3922 | Stoloniferin IV/ Stoloniferin V |
| **93** | 9.25 | 1413.7448 | C70H112O26 | M+FA-H | 1.7478 | Cairicoside B/ Cairicoside A/ Cairicoside IV |
| **94** | 9.27 | 1199.6191 | C55H94O25 | M+FA-H | 10.781 | Murucoidin VII |
| **95** | 9.28 | 1311.7347 | C63H110O25 | M-H, M+FA-H | 2.2427 | Cairicoside F/ Murucoidin VI |
| **96** | 9.29 | 1165.6740 | C57H100O21 | M-H, M+FA-H | 0.0334 | Murucoidin XIII/ Murucoidin XII |
| **97** | 9.3 | 1383.7409 | C69H110O25 | M+FA-H | 6.7904 | Batatoside P |
| **98** | 9.36 | 1251.7133 | C62H108O25 | M-H | 2.0412 | Stoloniferin VI |
| **99** | 9.38 | 1181.6677 | C58H102O24 | M-H | -0.9625 | Operculin XV/Batationoside IX/ Batatinoside VII/ Operculin XIII |
| **100** | 9.44 | 1237.7016 | C61H106O25 | M-H | 5.2856 | Multifidin II/ |
| **101** | 9.56 | 1413.7477 | C70H112O26 | M-H, M+FA-H | 3.8452 | Cairicoside IV/ Cairicoside A/ Cairicoside B |
| **102** | 9.71 | 1397.7542 | C70H112O25 | M+FA-H | 5.0065 | Pescaprein X/ Pescaprein XI/ Pescaprein XXIII/ Pescaprein XXIV |
| **103** | 9.71 | 1397.7542 | C70H112O25 | M+FA-H | 5.0065 | Pescaprein XVII / Pescaprein XVIII |
| **104** | 9.74 | 1067.5700 | C50H86O21 | M+FA-H | 7.7012 | Muricatin VII/ Tricolorin A/ Tricolorin D/ Tricolorin E |
| **105** | 9.74 | 1295.7391 | C63H110O24 | M-H, M+FA-H | 1.7580 | Batatinoside VIII/ Pescaprein III/ Stoloniferin X |
| **106** | 9.75 | 1149.6821 | C57H100O20 | M-H, M+FA-H | 2.7806 | Murucoidin XIV/ Pescaprein V |

| **107** | 9.76 | 1413.7485 | C70H112O26 | M+FA-H | 4.4702 | Leptophyllin A |
| --- | --- | --- | --- | --- | --- | --- |
| **108** | 9.82 | 1441.7764 | C72H116O26 | M-H, M+FA-H | 1.9608 | Batatoside I/Batatoside H |
| **109** | 9.87 | 1311.7349 | C63H110O25 | M-H, M+FA-H | 2.3911 | Simonin II |
| **110** | 9.87 | 1263.7436 | C64H112O24 | M-H | -2.7143 | Pescaprein IV/Pescaprein XX |
| **111** | 9.98 | 1279.7421 | C64H112O25 | M-H, M+FA-H | 0.0715 | Stoloniferin VII |
| **112** | 10.02 | 1311.7364 | C63H110O25 | M-H, M+FA-H | 3.6281 | Cairicoside F/ Murucoidin VI/ Quamoclin IV |
| **113** | 10.09 | 1427.7670 | C71H114O26 | M+FA-H | 6.5019 | Intrapilosin III |
| **114** | 10.16 | 1247.7922 | C64H114O20 | M+FA-H | 3.0208 | Operculin XII/ Operculin VI/ Operculin XI |
| **115** | 10.18 | 1425.7796 | C72H116O25 | M-H, M+FA-H | 0.5943 | Batatoside C/ Batatotinoside I/ Batatoside B/ Batatoside D/ Batatoside E/ Batatoside F |
| **116** | 10.25 | 1441.7800 | C72H116O26 | M-H, M+FA-H | 4.5262 | Intrapilosin IV/ Intrapilosin IV/ Intrapilosin V |
| **117** | 10.31 | 1425.7854 | C72H116O25 | M-H, M+FA-H | 4.8093 | Batatoside B/ Batatotinoside I/ Batatoside C/ Batatoside D/ Batatoside E/ Batatoside F |
| **118** | 10.38 | 1441.7799 | C72H116O26 | M-H, M+FA-H | 4.4721 | Batatoside H/ Batatoside I |
| **119** | 10.49 | 1353.7847 | C66H116O25 | M-H, M+FA-H | 4.5473 | Operculin II |
| **120** | 10.62 | 1197.6686 | C58H102O25 | M-H | 4.0412 | Operculin XVI/ Operculin XVIII |
| **121** | 10.9 | 1165.6746 | C57H100O21 | M+FA-H | 0.6138 | Murucoidin XII Murucoidin XIII/Pescaprein I |
| **122** | 10.9 | 1365.8135 | C68H120O24 | M-H, M+FA-H | -1.2197 | Simonin IV |
| **123** | 11.11 | 1381.8156 | C68H120O25 | M-H, M+FA-H | 4.1472 | Stoloniferin I/Operculin VII/ Operculin VIII |
| **124** | 11.12 | 1153.6416 | C56H98O24 | M-H | 3.4970 | Operculin XIV/Batatinoside VI |
| **125** | 11.13 | 981.4942 | C45H76O20 | M+FA-H | 3.1565 | Scammonin VI |
| **126** | 11.24 | 1231.7697 | C64H112O22 | M-H | 10.1142 | Marubajalapin IX/Marubajalapin VII/ Marubajalapin VIII/ Marubajalapin X/ Marubajalapin XI |
| **127** | 11.58 | 1247.7944 | C64H114O20 | M-H, M+FA-H | 4.8893 | Operculin VI/Operculin XI/ Operculin XII |
| **128** | 12.12 | 969.4939 | C44H76O20 | M+FA-H | 2.9416 | Scammonin V |

**Table S3**. Significant differential resin glycosides between *Beniazuma* samples and others

| Variable ID (RT_m/z) | Compound | VIP[1] | S-plot |
| --- | --- | --- | --- |
| 0.77_1035.5307m/z | Scammonin VIII | 1.0616 | B |
| 0.86_1019.5388m/z | Scammonin I/ Scammonin VII | 1.5366 | B |
| 4.05_1438.8302n | Intrapilosin VII | 1.0403 | B |
| 11.11_1336.8174n | Stoloniferin I/ Operculin VII/ Operculin VIII | 1.6434 | B |
| 2.98_1152.6370n | Murucoidin III | 1.5147 | T |
| 3.11_1213.6270m/z | Murucoidin V | 1.4066 | T |
| 5.12_1194.6791n | Murucoidin XVIII/ Stoloniferin VIII | 1.8362 | T |
| 5.61_1210.6740n | Multifidin I/ Quamoclin III | 5.5409 | T |
| 7.80_1222.7111n | Pescaprein XXX/ Stoloniferin III/ Stoloniferin IX | 1.2548 | T |
| 8.53_1385.7152m/z | Cairicoside D/ Cairicoside III/ Intrapilosin II | 2.0741 | T |
| 8.57_1238.7050n | Cairicoside E | 5.0518 | T |
| 9.25_1283.7047m/z | Stoloniferin IV/ Stoloniferin V | 1.222 | T |
| 9.30_1383.7409m/z | Batatoside P | 1.1951 | T |
| 9.76_1413.7485m/z | Leptophyllin A | 2.2196 | T |
| 9.87_1266.7366n | Simonin II | 1.0358 | T |
| 10.25_1396.7818n | Intrapilosin IV/ Intrapilosin VI/ Intrapilosin V | 3.2788 | T |
| 10.38_1396.7817n | Batatoside H/ Batatoside I | 3.0153 | T |
| 10.49_1308.7865n | Operculin II | 1.8372 | T |

B: left bottom, T: right top in S-plot

**Table S4.** Significant differential RGs between two *Beniazuma* samples.

| Variable ID (RT_m/z) | Compound | VIP[1] | S-plot |
| --- | --- | --- | --- |
| 0.72_1053.5565m/z | Tricolorin B | 1.0601 | B |
| 0.73_1051.5577m/z | Mammoside A/ Muticatin I | 1.0418 | B |
| 0.95_1179.6854m/z | Marubajalapin V/ Marubajalapin IV/Marubajalapin III | 1.6682 | B |
| 1.39_1133.6836m/z | Marubajalapin V/ Marubajalapin IV/ Marubajalapin III | 1.1476 | B |
| 2.91_1153.6310m/z | Operculin XIV/ Batatinoside VI | 1.065 | B |
| 3.02_1051.5691m/z | Muricatin I | 1.4267 | B |
| 3.88_1210.6745n | Quamoclin III/Multifidin I | 1.2248 | B |
| 4.03_1313.6641m/z | Batatoside A/Batataoside I/ Batataoside II | 1.1268 | B |
| 4.39_1343.6712m/z | Murucoidin XVII/ Cairicoside I | 1.049 | B |
| 5.12_1194.6791n | Murucoidin XVIII/Stoloniferin VIII | 2.2554 | B |
| 5.61_1210.6740n | Multifidin I/ Quamoclin III | 2.5482 | B |
| 5.73_1194.6780n | Stoloniferin VIII/ Murucoidin XVIII | 1.1998 | B |
| 6.35_1253.6932m/z | Stoloniferin II | 1.2475 | B |
| 7.53_1369.7216m/z | Pescaprein XXVIII | 1.3533 | B |
| 7.80_1222.7111n | Pescaprein XXX/Stoloniferin III/ Stoloniferin IX | 1.5763 | B |
| 8.53_1385.7152m/z | Cairicoside D/ Cairicoside III/ Intrapilosin II | 3.6925 | B |
| 8.54_1339.7108m/z | Cairicoside III/Cairicoside D | 1.4391 | B |
| 9.00_1383.7384m/z | Batatoside G | 1.5063 | B |
| 9.01_1238.7062n | Leptophyllin B | 1.5021 | B |
| 9.25_1283.7047m/z | Stoloniferin IV/Stoloniferin V | 1.6 | B |
| 9.25_1413.7448m/z | Cairicoside B/Cairicoside A/ Cairicoside IV | 1.0592 | B |
| 9.28_1266.7365n | Cairicoside F/ Murucoidin VI/ Murucoidin XI/ Quamoclin IV | 1.3247 | B |

| 9.29_1120.6757n | Murucoidin XIII/Murucoidin XII | 1.4531 | B |
| --- | --- | --- | --- |
| 9.30_1383.7409m/z | Batatoside P | 2.046 | B |
| 9.71_1397.7542m/z | Pescaprein XI | 1.0093 | B |
| 9.74_1250.7409n | Batatinoside VIII/Pescaprein III/ Stoloniferin X | 1.5283 | B |
| 9.75_1104.6839n | Murucoidin XIV/ Pescaprein V | 1.462 | B |
| 9.76_1413.7485m/z | Leptophyllin A | 1.568 | B |
| 9.82_1396.7782n | Batatoside I/ Batatoside H | 1.7319 | B |
| 9.98_1280.7494n | Stoloniferin VII | 1.6024 | B |
| 10.09_1427.7670m/z | Intrapilosin III | 1.7514 | B |
| 10.18_1380.7814n | Batatoside C/ Batatoside B/ Batatotinoside I/ Batatoside D/ Batatoside E/ Batatoside F | 1.5548 | B |
| 10.49_1308.7865n | Operculin II | 2.6847 | B |
| 11.11_1336.8174n | Stoloniferin I/ Operculin VII/ Operculin VIII | 1.5854 | B |
| 2.44_1183.6231m/z | Murucoidin IX/Murucoidin II | 1.6122 | T |
| 2.62_1183.6366m/z | Batatinoside V/Pescaprein VII/ Pescaperin VI | 2.068 | T |
| 2.72_1396.7782n | Batatoside M | 1.1258 | T |
| 12.12_969.4939m/z | Scammonin V | 1.0269 | T |

B: left bottom, T: right top in S-plot

**Table S5.** Significant differential RGs between Xu 32 samples and others (except Blackheart)

| Variable ID (RT_m/z) | Compound name | VIP[1] | S-plot |
| --- | --- | --- | --- |
| 3.63_1213.6298m/z | Murucoidin IV | 1.13364 | B |
| 5.61_1210.6740n | Multifidin I/Quamoclin III | 1.15676 | B |
| 6.19_1210.6773n | Quamoclin III/ Multifidin I | 2.09682 | B |
| 8.53_1385.7152m/z | Cairicoside D/ Cairicoside III | 1.61142 | B |
| 9.01_1238.7062n | Leptophyllin B | 1.68199 | B |
| 9.76_1413.7485m/z | Leptophyllin A | 1.18538 | B |
| 2.37_1425.7811m/z | Pescaprein XXI/ Pescaprein XXII | 1.58264 | T |
| 2.47_1425.7797m/z | Pescaprein XXI/ Pescaprein XXII | 1.5366 | T |
| 2.72_1396.7782n | Batatoside M | 1.19178 | T |
| 2.98_1152.6370n | Murucoidin III | 1.03497 | T |
| 2.99_1352.8084n | Operculin IX/Operculin X | 1.79026 | T |
| 5.12_1194.6791n | Murucoidin XVIII/ Stoloniferin VIII | 2.5675 | T |
| 5.85_1320.7987n | Batatoside J | 1.79064 | T |
| 6.26_1364.8412n | Operculin I/ Operculin V | 1.39555 | T |
| 6.35_1253.6932m/z | Stoloniferin II | 1.17191 | T |
| 7.80_1222.7111n | Pescaprein XXX/ Stoloniferin III/ Stoloniferin IX | 3.04301 | T |
| 8.96_1227.6796m/z | Batatinoside IX/ Batatinoside VII/ Operculin XIII | 1.74311 | T |
| 9.28_1266.7365n | Cairicoside F/ Murucoidin VI/ Murucoidin XI/ Quamoclin IV | 3.68042 | T |
| 9.72_1281.7217m/z | Pescaprein XVII/ Pescaprein XVIII | 1.45227 | T |
| 9.74_1250.7409n | Batatinoside VIII/ Pescaprein III/ Stoloniferin X | 3.66049 | T |
| 9.87_1266.7366n | Simonin II | 1.23042 | T |
| 10.18_1380.7814n | Batatoside C/ Batatoside B/ Batatoside D/ Batatoside E/ Batatoside F/Batatotinoside I | 1.64464 | T |
| 10.31_1380.7872n | Batatoside B/ Batatoside C/ Batatoside D/ Batatoside E/ Batatoside F/Batatotinoside I | 1.48696 | T |
| 10.90_1320.8153n | Simonin IV | 2.68426 | T |
| 11.11_1336.8174n | Stoloniferin I/ Operculin VII/ Operculin VIII | 4.4363 | T |

10

**Table S6.** Significant differential RGs between Blackheart samples and others (except Xu 32)

| Variable ID (RT_m/z) | Accepted ID | VIP[1] | S-plot |
| --- | --- | --- | --- |
| 2.44_1183.6231m/z | Murucoidin IX/Murucoidin II | 1.57783 | B |
| 2.62_1183.6366m/z | Batatinoside V/Pescaprein VI/ Batatinoside V | 2.15801 | B |
| 2.98_1152.6370n | Murucoidin III | 1.24507 | B |
| 5.12_1194.6791n | Murucoidin XVIII/Stoloniferin VIII | 1.26282 | B |
| 5.61_1210.6740n | Multifidin I/ Quamoclin III | 2.56848 | B |
| 7.80_1222.7111n | Pescaprein XXX/ Stoloniferin III/ Stoloniferin IX | 1.08502 | B |
| 8.57_1238.7050n | Cairicoside E | 2.48442 | B |
| 0.76_753.3850m/z | Tricolorin F | 1.13838 | T |
| 0.77_1035.5307m/z | Scammonin VIII | 2.13355 | T |
| 0.78_1017.5210m/z | Scammonin IV | 1.67948 | T |
| 0.86_1019.5388m/z | Scammonin I/ Scammonin VII | 3.5878 | T |
| 12.12_969.4939m/z | Scammonin V | 1.3381 | T |
| 2.72_1396.7782n | Batatoside M | 1.17515 | T |
| 3.46_1343.6685m/z | Cairicoside I/ Cairicoside C/ Intrapilosin I/ Murucoidin XVII | 1.635 | T |
| 3.70_1365.7974m/z | Batatoside J | 1.88839 | T |
| 4.03_1313.6641m/z | Batataoside I/ Batataoside II/ Batatoside A | 1.68374 | T |
| 4.68_1019.6168m/z | Batatinoside III | 1.96136 | T |
| 5.19_1298.6738n | Cairicoside I/ Cairicoside C/ Intrapilosin I/ Murucoidin XVII | 1.28722 | T |
| 8.53_1385.7152m/z | Cairicoside D/Cairicoside III/ Intrapilosin II | 1.06685 | T |
| 9.00_1383.7384m/z | Batatoside G | 1.86085 | T |
| 9.01_1238.7062n | Leptophyllin B | 1.49798 | T |
| 9.28_1266.7365n | Cairicoside F/Murucoidin VI/ Murucoidin XI/ Quamoclin IV | 4.37072 | T |
| 9.74_1250.7409n | Batatinoside VIII/Stoloniferin X | 3.68624 | T |
| 9.87_1266.7366n | Simonin II | 1.84212 | T |

**
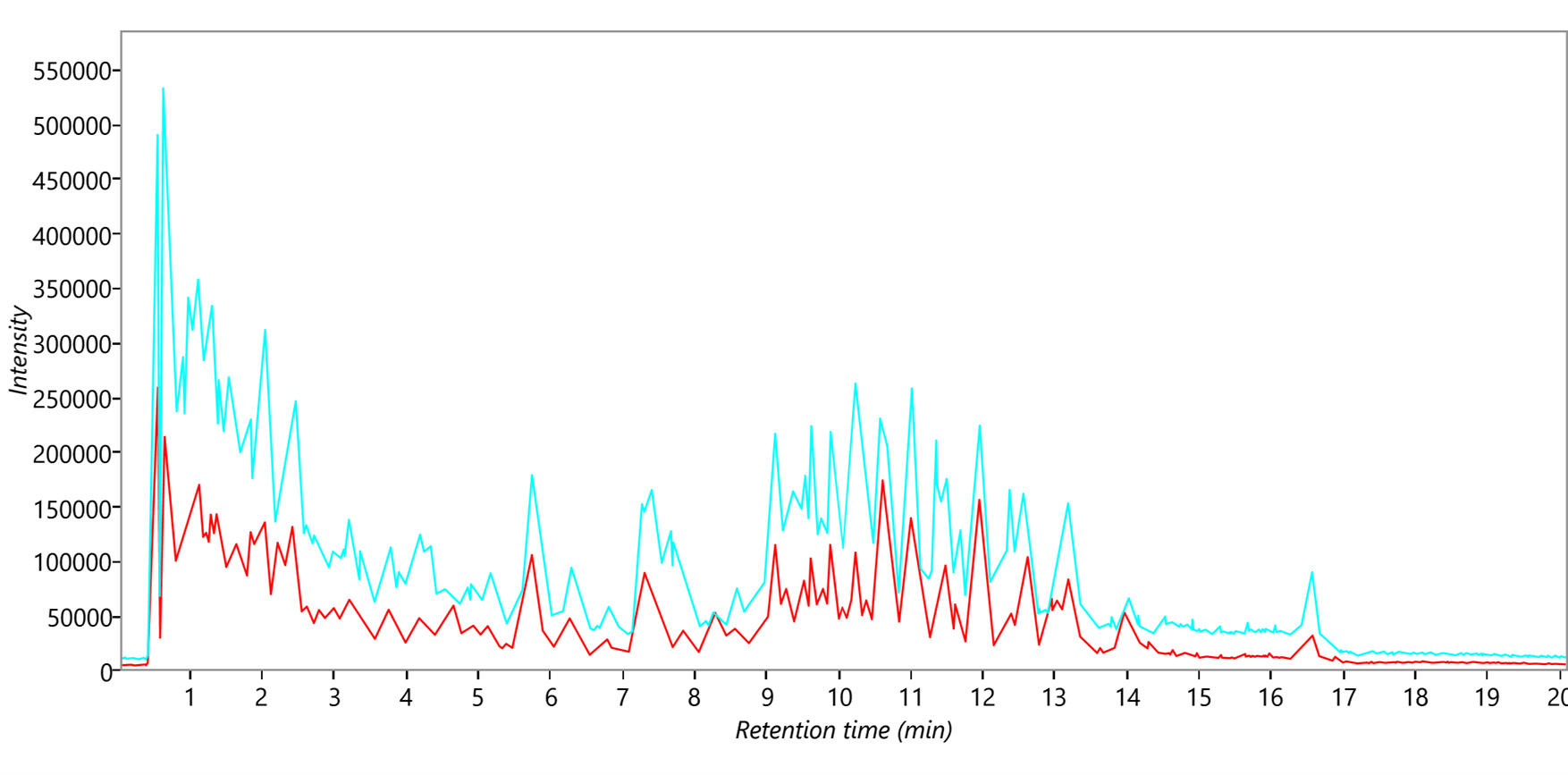
Figure S1** Representative total ion chromatogram (TIC) of the quality control (QC) samples in positive and negative ion modes. A quality control (QC) sample was prepared by pooling equal aliquots of resin glycoside extracts from all selected cultivars.
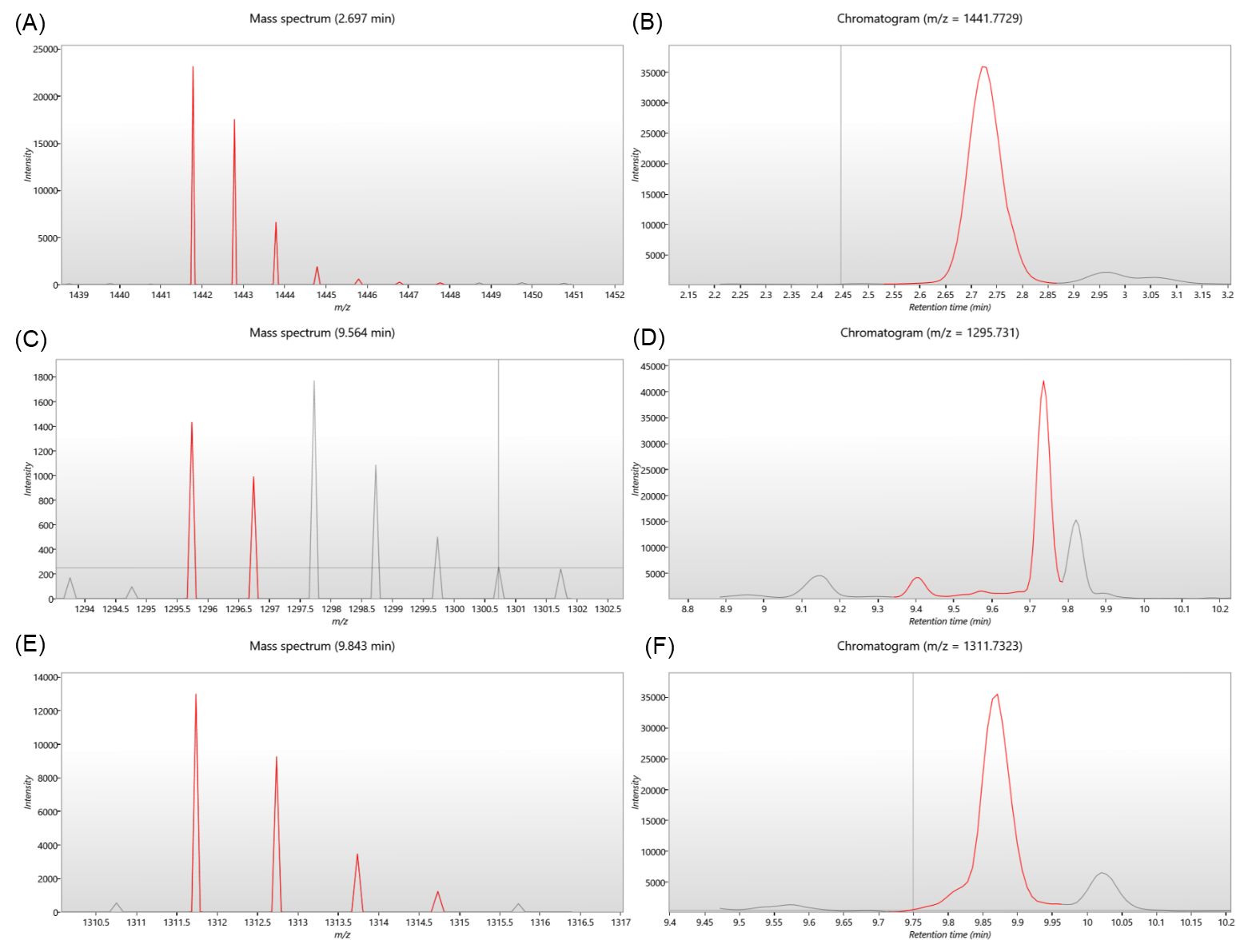


**Figure** **S2**. Representative mass spectra (MS) and corresponding extracted ion chromatograms (XICs) of resin glycosides: MS (A) and XIC (B) of batatoside M; MS (C) and XIC (D) of cairicoside F/ murucoidin VI; MS (E) and XIC (F) of simonin II.


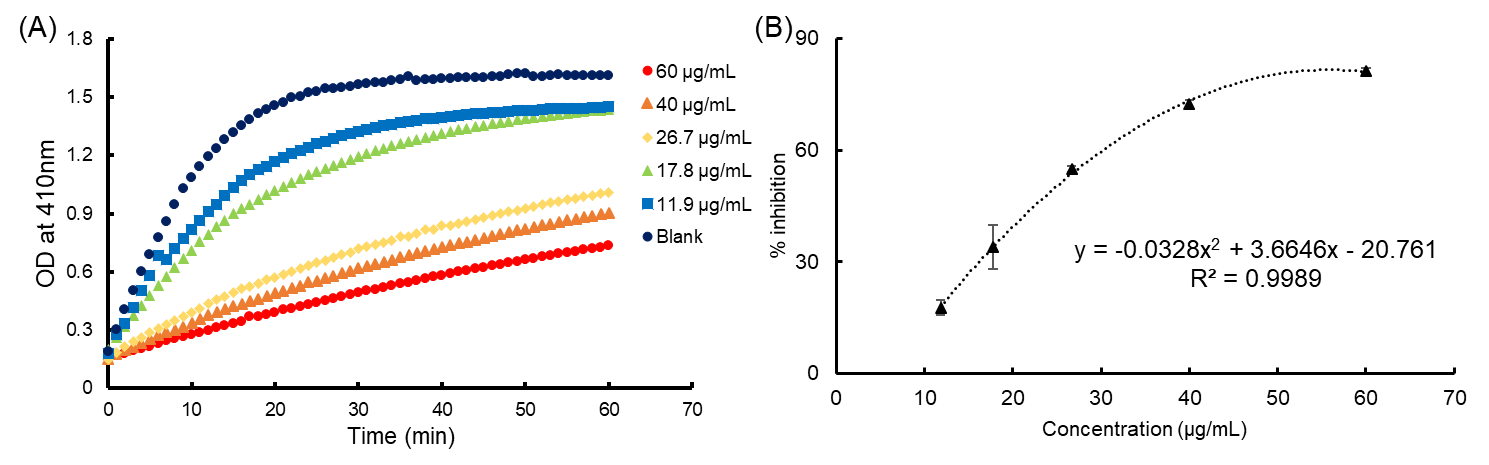


**Figure S3** (A) Kinetic curves of optical density at 410 nm under different concentrations of Xu32 extract in the presence of pNPP (B) Dose–response curves of Xu32 extracts in pancreatic lipase inhibition.


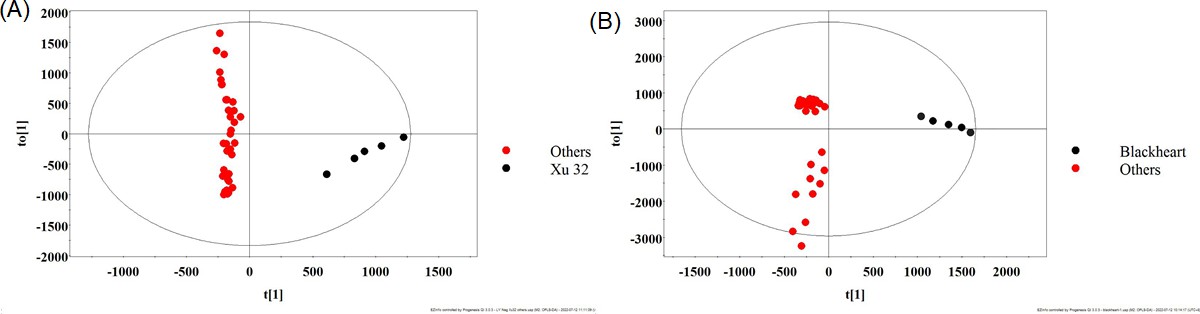


**Figure S4.** OPLS-DA score plot of RG (A) Xu32 *vs* Others (B) Blackheart *vs* Others.


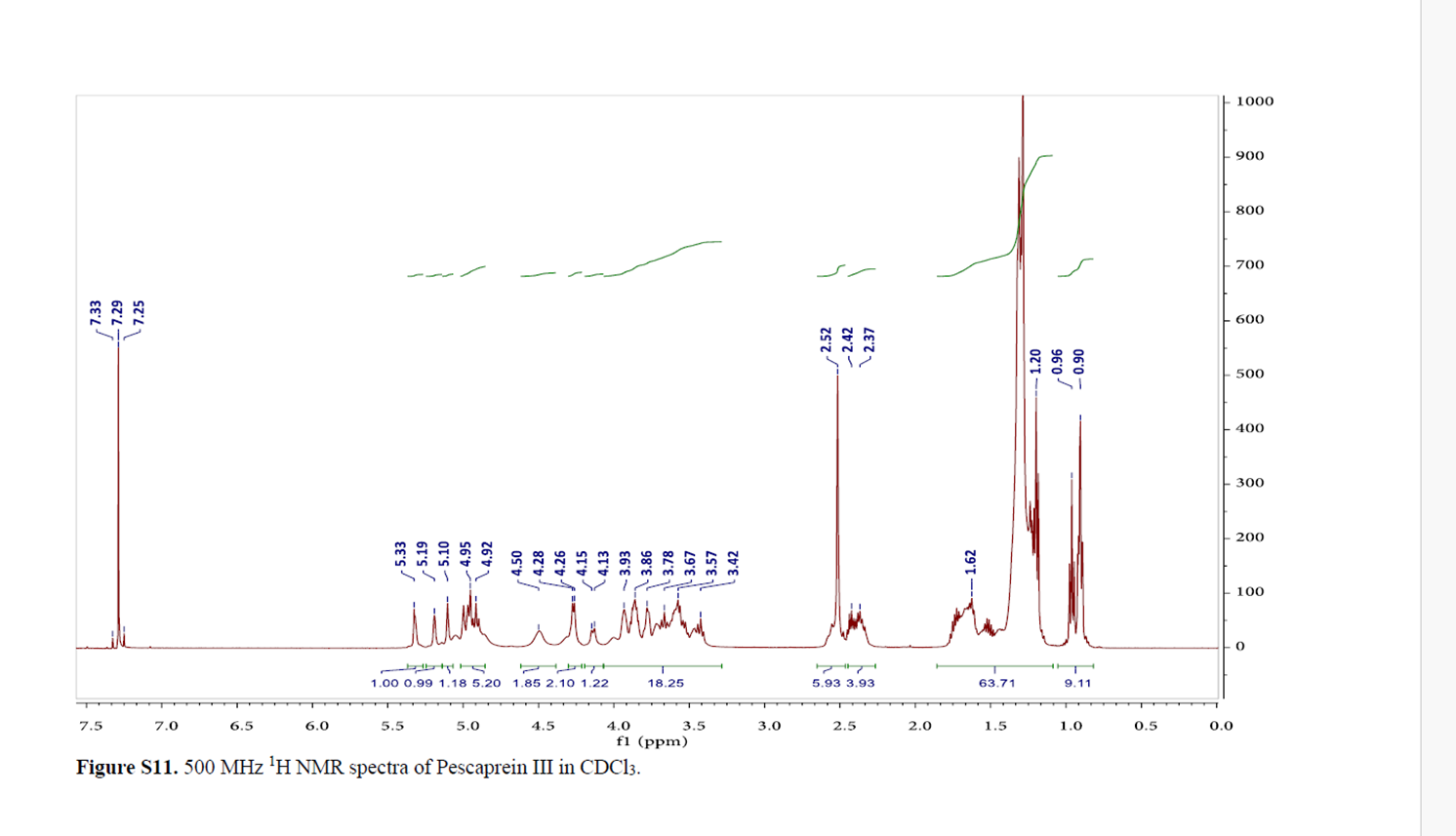


**Figure S5**. 500 MHz ^1^H NMR spectra of Pescaprein III in CDCl_3_.


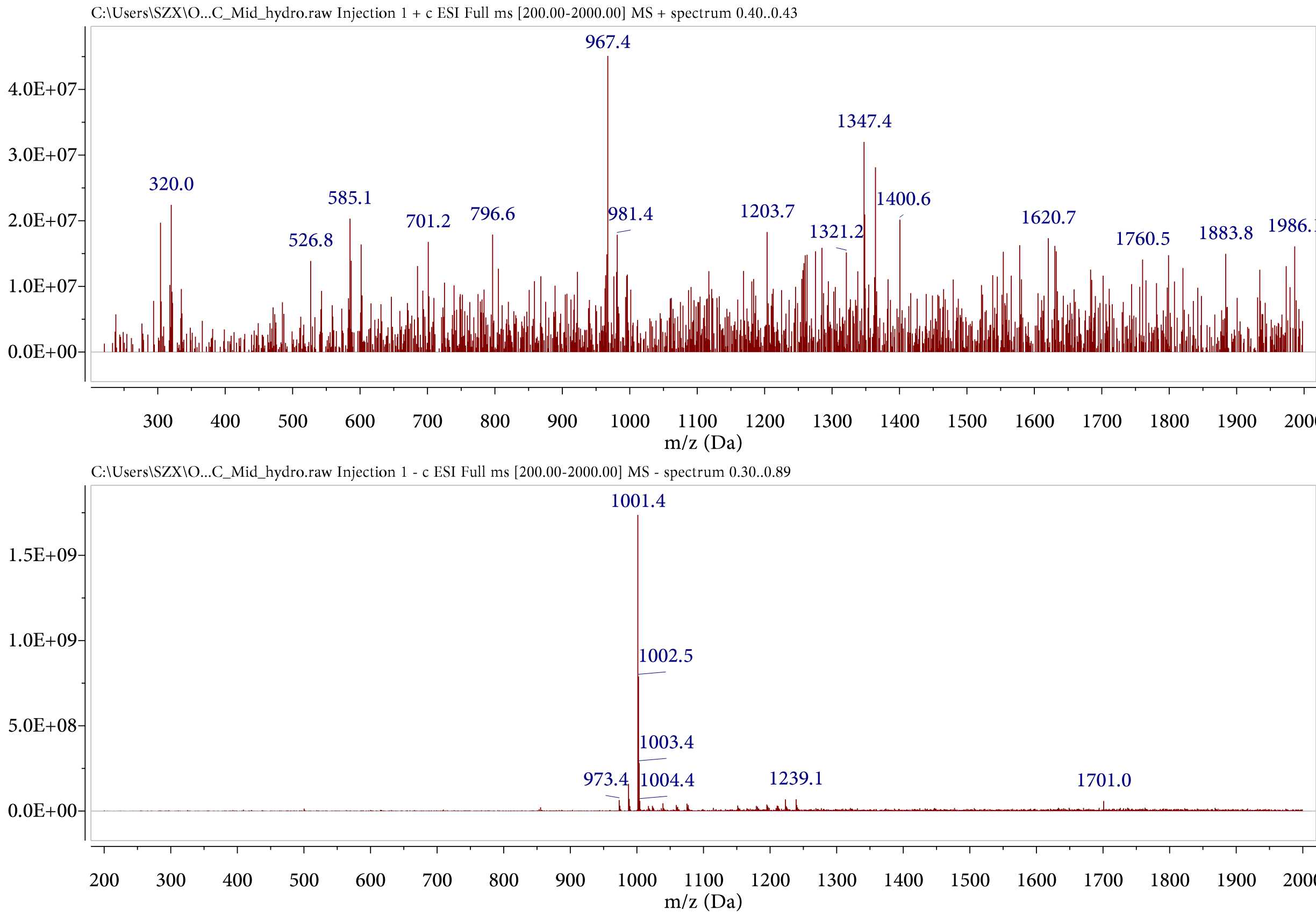


**Figure S6.** Mass spectrum of simonic acid B
